# A Mathematical Analysis of Aerobic Glycolysis Triggered by Glucose Uptake in Cones

**DOI:** 10.1038/s41598-019-39901-z

**Published:** 2019-03-11

**Authors:** Erika T. Camacho, Danielle Brager, Ghizlane Elachouri, Tatyana Korneyeva, Géraldine Millet-Puel, José-Alain Sahel, Thierry Léveillard

**Affiliations:** 1 School of Mathematical & Natural Sciences, Arizona State University, Glendale, AZ 85306 USA; 2 School of Mathematical & Statistical Sciences, Arizona State University, Tempe, AZ 85287 USA; 3 Institut de la Vision: Department of genetics Sorbonne Université, INSERM, CNRS, Institut de la Vision, 17 rue Moreau, F-75012 Paris, France

## Abstract

Patients affected by retinitis pigmentosa, an inherited retinal disease, experience a decline in vision due to photoreceptor degeneration leading to irreversible blindness. Rod-derived cone viability factor (RdCVF) is the most promising mutation-independent treatment today. To identify pathologic processes leading to secondary cone photoreceptor dysfunction triggering central vision loss of these patients, we model the stimulation by RdCVF of glucose uptake in cones and glucose metabolism by aerobic glycolysis. We develop a nonlinear system of enzymatic functions and differential equations to mathematically model molecular and cellular interactions in a cone. We use uncertainty and sensitivity analysis to identify processes that have the largest effect on the system and their timeframes. We consider the case of a healthy cone, a cone with low levels of glucose, and a cone with low and no RdCVF. The three key processes identified are metabolism of fructose-1,6-bisphosphate, production of glycerol-3-phosphate and competition that rods exert on cone resources. The first two processes are proportional to the partition of the carbon flux between glycolysis and the pentose phosphate pathway or the Kennedy pathway, respectively. The last process is the rods’ competition for glucose, which may explain why rods also provide the RdCVF signal to compensate.

## Introduction

Rods and cones are photoreceptor cells located in the retina, which play a central role in the vision process. Light photons are detected by the photoreceptors and processed into electrical signals in the retina. In mammals, rods are much more numerous than cones existing at a ratio of about 20 rods per cone. The rods are responsible for night vision while the cones are responsible for color vision and visual acuity. The photoreceptors are the most metabolically demanding cells in the body and are in constant need of nutrients, glucose, lipids, and metabolites for maintenance^1^. As part of that maintenance, the photoreceptors undergo renewal and periodic shedding of their outer segment (OS) discs to prevent the toxic effects of accumulated photo-oxidative products. Following shedding, photoreceptors regenerate about the same amount of cellular material each day maintaining a relatively constant length of their outer segments^2,3^.

When photoreceptor degeneration occurs, rod and cone OS begin to shorten as a result of disruptions in the renewal and underlying metabolic processes. These disruptions, if magnified, can lead to eventual death of the photoreceptors. In the mature human retina (by about age 5 or 6), there are no spontaneous births of photoreceptors. Thus, when a photoreceptor dies, there is no new photoreceptor created. As photoreceptor death continues due to degeneration, loss of vision progresses resulting in blindness. Apart from anti vascular endothelial growth factor (anti-VEGF) medications that can limit progression of choroidal neovascularisation, a clinical form of age-related macular degeneration, there are no cures for diseases, such as retinitis pigmentosa (RP), that are linked to photoreceptor degeneration^4^. Typically, RP is characterized by the death of rods due to some genetic mutation followed by the death of cones. The peculiarity is that the cones die after the rods even if the cones are genetically healthy.

Understanding what caused the secondary wave of cone death following rod degeneration in RP was for many years the driving force behind numerous studies. In 2004, Léveillard *et al*. identified and characterized a protein they coined the rod-derived cone viability factor (RdCVF). Their experiments showed that RdCVF significantly preserves cone function and vitality. A 40% rescue effect in the presence of RdCVF was observed experimentally and confirmed *in silico*^5,6^. Understanding the mechanisms by which RdCVF preserves cones’ function is essential because maintaining functional cones even when 95% are gone may prevent blindness^7,8^.

We know that RdCVF promotes cone survival by stimulating aerobic glycolysis in cones. The process of aerobic glycolysis allows for energy production and phospholipid synthesis, which is needed for the renewal of cone OS^9,10^. Basigin-1 (BSG1) forms a complex, GLUT1/BSG1, with glucose transporter 1 (GLUT1, SLC2A1). RdCVF triggers aerobic glycolysis by binding to the GLUT1/BSG1 complex and accelerates the entry of glucose into the cell. Using a model of aerobic glycolysis in a single cone cell embedded in a cell population, we investigate the interrelated and feedback dynamics from the molecular to the population level and vice versa. The population component describes the dynamics at the cellular level, and it has been extensively developed and analyzed^11–13^. We model explicitly the mode of action of RdCVF and glucose uptake with the goal of understanding the key mechanisms that drive cone OS renewal in a healthy retina so that these conclusions may guide experimental work and identify key processes. The purpose of this study is to mathematically analyze the molecular and cellular level interactions that occur in a non-diseased retina with the goal of gaining insight into an understanding of photoreceptor vitality so that our findings may contribute to the development of therapies that can stop cone photoreceptor degeneration.

## Results

### Modeling Reaction Rates

**Figure 1:**
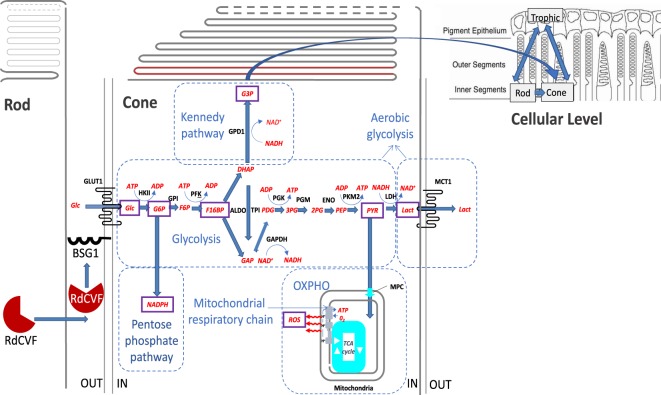
Molecular steps modeled in aerobic glycolysis. The biochemical quantities we model in the system are boxed in or circled. This picture has been modified from^9,10^.

In our mathematical model, represented by a set of nonlinear ordinary differential equations (ODEs) in section 4.1, we focus on the critical metabolic steps triggered by the uptake of glucose into the cones. We model three phases of glucose catabolism and their interplay in order to better illustrate the mediated survival exerted by the rods on the cones. The three metabolic phases include aerobic glycolysis, oxidative phosphorylation (OXPHO), and the pentose phosphate pathway (PPP); see Fig. 1. The term aerobic glycolysis means that glucose produces lactate even in the presence of oxygen. Cone photoreceptors will not survive without mitochondria and oxygen. There is a partitioning in the cone photoreceptors between glucose going through aerobic glycolysis for cone OS renewal and glucose going through OXPHO, the main adenosine triphosphate (ATP) production step. RdCVF stimulates glucose going through aerobic glycolysis, while reactive oxygen species (ROS) favors glucose leading to the production of carbon dioxide (CO_2_) and the reduced form of nicotinamide adenine dinucleotide phosphate (NADPH), CO_2_ + NADPH, through the PPP and inhibits both glucose flowing through aerobic glycolysis and OXPHO. In the case of too much ROS, the requirement for more NADPH to cope with the stress results in the oxidation of all 6 carbon atoms of glucose into 6 CO_2_ molecules through the PPP. In healthy conditions, ROS is a function of glucose metabolized through OXPHO and the percent of leakage of the mitochondrial respiratory chain.

Our model does not include a complete mathematical representation of OXPHO and the PPP. Currently, certain processes in these metabolic phases are not completely known; therefore, there is no way to obtain certain measurements necessary to validate a model consisting of a complete representation of all the reactions of these phases. Thus in considering the PPP and OXPHO, we focus on the mechanisms affecting ROS inhibition or production which are key to cones’ vitality and for which we have a better handle on particular quantities.

We incorporate the dominant divergence of pyruvate into this pathway, and a means to create ROS through the leakage in the mitochondrial respiratory chain^14^. We also illustrate the detoxification of ROS that results from the production of NADPH through the PPP by including an equation for the reaction rate of NADPH production^15^. Glucagon is not included in our model. This peptide hormone produced by pancreatic cells raises the concentration of glucose in the bloodstream. The expression of the glucagon-like peptide-1 receptor (GLP1R) is downregulated in the retinal pigment epithelium (RPE) in a model of chemically induced diabetic retinopathy^16^. Since this phenomenon is taking place at the opposite side of the outer blood retinal barrier, the basal side of the RPE, we do not include glucagon as an inhibitor of glycolysis. Additionally, we were unable to find data showing that cone photoreceptors express GLP1R.

We model aerobic glycolysis starting with the binding of RdCVF to the BSG1/GLUT1 complex and ending with production of lactate (LACT). The carbon flux is diverted from aerobic glycolysis at some specific point in the reaction and the 6 carbons of glucose are not entirely metabolized into 2 molecules of lactate (3 carbons) so that an unknown proportion of carbon is transferred from glucose to phospholipids to form the cone OS. In our mathematical model, we will take this into account in the amount of glucose the cones uptake to produce new cone OS. We let [*δR*_*n*_] represent the free unbound RdCVF concentration which eventually binds to the BSG1/GLUT1 complex to activate GLUT1 and initiate the biochemical cascade leading to the production of LACT as well as the production of ROS through OXPHO.

Our mathematical model consists of a system of nonlinear ODEs that describes the molecular and cellular level photoreceptor interactions. Below, we first consider the reaction rate equations for for eight biochemical molecules (see Fig. 1), and then use those equations to construct the molecular level ODEs; see Section 2.2.

Considering *δR*_*n*_ to be the substrate, *g* the glucose inside the cell, and *G* the glucose outside the cell, in the chemical transformation of active GLUT1, we have the chemical reactions

$$[\delta {{\rm{R}}}_{{\rm{n}}}]+[{\rm{BSG}}1/\mathrm{GLUT}1]\leftrightharpoons [\delta {{\rm{R}}}_{{\rm{n}}}/\mathrm{BSG}1/\mathrm{GLUT}1]$$

$${\rm{and}}$$

$$[{\rm{G}}]+[\delta {{\rm{R}}}_{{\rm{n}}}/\mathrm{BSG}1/\mathrm{GLUT}1]\to [{\rm{g}}]+[\delta {{\rm{R}}}_{{\rm{n}}}/\mathrm{BSG}1/\mathrm{GLUT}1].$$

When the 3-protein complex *δR*_*n*_/BSG1/GLUT1 is formed, an allosteric regulation leads to the activation of GLUT1, a facilitated transporter^15^. The only backward reaction is the dissociation of RdCVF and BSG1/GLUT1. The reaction then goes only forward. In this allosteric regulation, RdCVF stimulates the transport activity of GLUT1 by triggering its tetramerization which relies on a reversible redox-dependent interconversion. Accessible cysteine residues in GLUT1 would be oxidized by the extracellular and oxidized form of RdCVF (RdCVF^ox^) that would act as a prooxidant. Oxidized thioredoxins are prooxidant as is the protein disulfide isomerase, another thioredoxin enzyme that catalyzes the formation of disulfide bridges (oxidation of two cysteines) in proteins transiting through the endoplasmic reticulum^13,17,18^. GLUT1 accelerates the rate of glucose intake by cones. It does not require energy for transporting glucose and the rate of transport is a function of the gradient of the concentration of glucose outside the cell, [*G*], versus the concentration of glucose inside the cell, [g] and the limiting value of the transport rate of glucose, represented by ${V}_{{{\rm{\max }}}_{[{\rm{g}}]}}$. Given this, at a certain glucose concentration in the retina (interphotoreceptor space), the amount of glucose transport depends on the rate of metabolism of glucose by the respective photoreceptors. The cell viability, which depends on glucose uptake and metabolism through aerobic glycolysis, is stimulated by RdCVF^10^. We model the effects of these chemical reactions that accelerate the rate of glucose intake by cones as

$${\nu }_{[{\rm{g}}]}=\lambda ([G]-[{\rm{g}}])\,(\frac{{V}_{{{\rm{\max }}}_{[{\rm{g}}]}}{(\delta {R}_{n})}^{2}}{{K}_{{m}_{[{\rm{g}}]}}^{2}+{(\delta {R}_{n})}^{2}}+p)$$

where [*G*], which is between 3.3 to 7 mM, represents the total glucose concentration outside the cell, [g] is the glucose concentration inside the cell, ${V}_{{{\rm{\max }}}_{[{\rm{g}}]}}$ is the limiting value (i.e., the saturation value) of the transport rate of glucose, *λ* is a conversion factor, and *p* is the glucose uptake of cones in the absence of RdCVF. The parameter ${K}_{{m}_{[{\rm{g}}]}}$ is equal to the substrate concentration that gives half the limiting value of the transport rate of glucose, ${V}_{{{\rm{\max }}}_{[{\rm{g}}]}}$. Experimental work of ^10^ indicated that $\frac{{V}_{{{\rm{\max }}}_{[{\rm{g}}]}}{(\delta {R}_{n})}^{2}}{{K}_{{m}_{[{\rm{g}}]}}^{2}+{(\delta {R}_{n})}^{2}}\gg p$ where $\frac{{V}_{{{\rm{\max }}}_{[{\rm{g}}]}}{(\delta {R}_{n})}^{2}}{{K}_{{m}_{[{\rm{g}}]}}^{2}+{(\delta {R}_{n})}^{2}}$ represents the glucose uptake in the presence of RdCVF. When there is an allosteric regulation in the production [i], there is a binding time requirement for the enzyme to catalyze the formation of the product. In this case, the plot of each ${\nu }_{[i]}$ vs. [*i*] would be sigmoidal. To model this mathematically, we use a Holling’s type III functional response equation instead of the Holling’s type II functional response which is used in the formulation of the Michaelis-Menten equation.

The next committed step we model is the production of glucose-6-phosphate (G6P), where *g* is the substrate and hexokinase 2 (HKII) is the enzyme in the reaction,

$$[{\rm{g}}]+[{\rm{HKII}}]\leftrightharpoons [g/\mathrm{HKII}]\to [{\rm{G}}6{\rm{P}}]+[{\rm{HKII}}].$$

The irreversible step in this reaction is the formation of G6P: the conversion of glucose to G6P catalyzed by the enzyme hexokinase 2 requires ATP as a high-energy phosphoryl donor. Phosphorylation of the primary hydroxyl group at C-6 yields a negatively charged derivative whose phosphate group will be used to generate ATP from adenosine diphosphate (ADP) later in the pathway^19^. Phosphorylation also prevents glucose from escaping from the cell cytosol. The reaction rate of G6P is governed by

$${\nu }_{[{\rm{G}}6{\rm{P}}]}=\frac{{V}_{{{\rm{\max }}}_{[{\rm{G}}6{\rm{P}}]}}{[{\rm{g}}]}^{2}}{{K}_{{m}_{[{\rm{G}}6{\rm{P}}]}}^{2}+{[{\rm{g}}]}^{2}},$$

where [G6P] is the concentration of G6P, ${V}_{{{\rm{\max }}}_{[\mathrm{G6P}]}}$ is the limiting value of the reaction rate of G6P, and ${K}_{{m}_{[{\rm{G6P}}]}}$ is the substrate concentration that gives half the maximal rate.

We will assume the intermediate step between G6P and fructose-6-phosphate (F6P) takes place. This assumption allows us to move on and model the production of fructose-1,6-bisphosphate (F16BP), the next committed step of glycolysis. We let G6P be the substrate and phosphofructokinase (PFK) be the enzyme in the reaction rate of F16BP such that

$$[{\rm{G}}6{\rm{P}}]+[{\rm{PFK}}]\leftrightharpoons [{\rm{G}}6P/\mathrm{PFK}]\to [{\rm{F}}16{\rm{BP}}]+[{\rm{PFK}}].$$

This chemical reaction is governed by

1 $${\nu }_{[{\rm{F}}16{\rm{BP}}]}=(\frac{{V}_{{{\rm{\max }}}_{[{\rm{F}}16\mathrm{BP}]}}{[{\rm{G}}6{\rm{P}}]}^{2}}{{K}_{{m}_{[{\rm{F}}16{\rm{BP}}]}}^{2}+{[{\rm{G}}6{\rm{P}}]}^{2}})\,{{\rm{\Omega }}}_{[\mathrm{PYR}]},$$

where

$${{\rm{\Omega }}}_{[{\rm{PYR}}]}=\frac{{[{\rm{PEP}}]}_{eq}^{4}}{{[{\rm{PEP}}]}_{eq}^{4}+{[{\rm{PYR}}]}^{4}},$$

${V}_{{{\rm{\max }}}_{[{\rm{F}}16{\rm{BP}}]}}$ is the limiting value of the reaction rate of F16BP, ${K}_{{m}_{[{\rm{F}}16{\rm{BP}}]}}$ is the substrate concentration that gives half the maximal rate, $[\mathrm{F16BP}]$ is the concentration of F16BP, $[{\rm{PYR}}]$ represents pyruvate (PYR) concentration, and ${[{\rm{PEP}}]}_{eq}$ is the switch that redirects glucose either to the PPP or down the aerobic glycolysis pathway to produce lactate.

Since we are not modeling all intermediate chemical reactions involved in aerobic glycolysis, we take ${[{\rm{PEP}}]}_{eq}$ in relation to $[{\rm{PYR}}]$ as a proxy for accumulation of phosphoenol pyruvate (PEP) due to oxidation of cysteine (358), a residue on pyruvate kinase. Therefore, we have ${[{\rm{PEP}}]}_{eq}$ explicit in equation (1). For $[{\rm{PYR}}]$ levels below ${[{\rm{PEP}}]}_{eq}$, G6P leads to an increase in PYR and the production of lactate through various biochemical steps in glycolysis. When $[{\rm{PYR}}] < {[{\rm{PEP}}]}_{eq}$, G6P encourages the production of F16BP. This process is illustrated by ${{\rm{\Omega }}}_{[{\rm{PYR}}]}$ in equation (1). When $[{\rm{PYR}}] > {[{\rm{PEP}}]}_{eq}$ the production of F16BP is inhibited and G6P diverts glucose into the PPP. The inclusion of ${[{\rm{PEP}}]}_{eq}$ redirects glucose into the PPP as illustrated with ${{\rm{\Phi }}}_{[{\rm{PYR}}]}$ in equation (2) below. Thus, in our mathematical model $[{\rm{PYR}}]$ together with ${[{\rm{PEP}}]}_{eq}$ serve a dual role regulating PYR production: acting as a proxy of glycolysis rate and the initiation of PPP.

Glucose metabolism through the PPP reduces nicotinamide adenine dinucleotide phosphate (NADP^+^) to NADPH. A first molecule of NADPH results from the action of the glucose-6-phosphate dehydrogenase by reducing one molecule of NADP^+^. A second molecule of NADPH results from the action of the 6-phosphogluconate dehydrogenase by reducing a second molecule of NADP^+^. The resulting 2 moles of NADPH allow for the detoxification of ROS and repair of oxidative damage in cones^15^. We incorporate the production of NADPH into our model by the following equation,

2 $${\nu }_{[{\rm{NADPH}}]}=(\frac{2{V}_{{{\rm{\max }}}_{[{\rm{NADPH}}]}}{[{\rm{G6P}}]}^{2}}{{K}_{{m}_{[{\rm{NADPH}}]}}^{2}+{[{\rm{G}}6{\rm{P}}]}^{2}})\,{{\rm{\Phi }}}_{[{\rm{PYR}}]}$$

where

$${{\rm{\Phi }}}_{[{\rm{PYR}}]}=\frac{{[{\rm{PYR}}]}^{4}}{{[{\rm{PEP}}]}_{eq}^{4}+{[{\rm{PYR}}]}^{4}},$$

**Figure 2:**
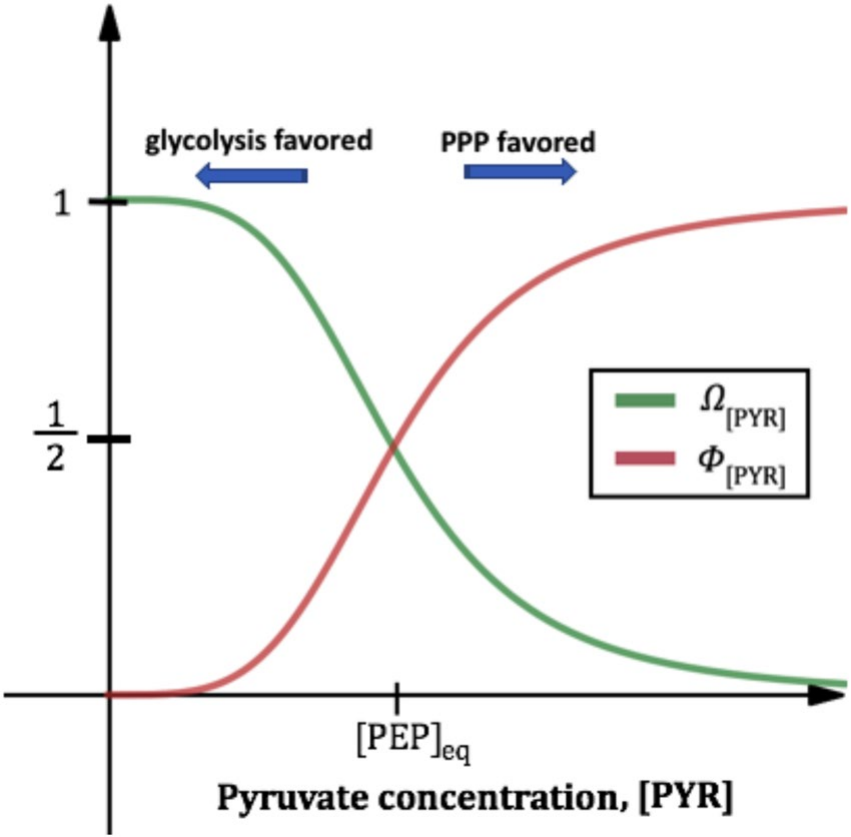
The rerouting of glucose is not an ON/OFF switch but rather a tuning switch as depicted by the graphs of ${{\rm{\Omega }}}_{[{\rm{PYR}}]}=\frac{{[{\rm{PEP}}]}_{eq}^{4}}{{[{\rm{PEP}}]}_{eq}^{4}+{[{\rm{PYR}}]}^{4}}$ and ${{\rm{\Phi }}}_{[{\rm{PYR}}]}=\frac{{[{\rm{PYR}}]}^{4}}{{[{\rm{PEP}}]}_{eq}^{4}+{[{\rm{PYR}}]}^{4}}$.

${V}_{{{\rm{\max }}}_{[{\rm{NADPH}}]}}$ is the limiting value of the reaction rate of NADPH, $[{\rm{NADPH}}]$ is the concentration of NADPH, and ${K}_{{m}_{[{\rm{NADPH}}]}}$ is the substrate concentration that gives half the maximal rate. The parameter ${[{\rm{PEP}}]}_{eq}$ is the critical level of pyruvate necessary to initiate the PPP. The graphs of ${{\rm{\Omega }}}_{[{\rm{PYR}}]}$ and ${{\rm{\Phi }}}_{[{\rm{PYR}}]}$ are shown in Fig. 2 below.

Branching from aerobic glycolysis, dihydroxyacetone phosphate (DHAP) is providing triglycerides via glycerol-3-phosphate (G3P), the precursors of phospholipids that are necessary for OS renewal. A portion of F16BP, *q*, is converted from DHAP to G3P and the remaining portion of F16BP, $(1\,-\,q)$, is converted from DHAP back to glyceraldehyde-3-phosphate (GAP). Aldolase splits one 6-carbon molecule of F16BP into two 3-carbon molecules: GAP and DHAP. GAP is entirely converted into lactate and CO_2_ by aerobic glycolysis and OXPHO, while a portion of DHAP is used for G3P and consequently cone OS renewal. Thus, *q* is proportional to the activity of *V*_max_ and *K*_*m*_ of triose phosphate isomerase (TPI). It is also the case that TPI activity is inhibited by PEP^20^ which accumulates in the presence of ROS.

Thus for the production of G3P, we take F16BP as a substrate, and cytoplasmic glycerol-3P-dehydrogenase (GPD1) as the enzyme in the production of G3P, such that

$$[{\rm{F}}16{\rm{BP}}]+[{\rm{GPD}}1]\leftrightharpoons [{\rm{F}}16\mathrm{BP}/\mathrm{GPD}1]\to [{\rm{G}}3{\rm{P}}]+[{\rm{GPD}}1].$$

The reaction rate of G3P in the model is governed by

$${\nu }_{[{\rm{G}}3{\rm{P}}]}=\frac{q{V}_{{{\rm{\max }}}_{[{\rm{G}}3{\rm{P}}]}}{[{\rm{F}}16{\rm{BP}}]}^{2}}{{K}_{{m}_{[{\rm{G3P}}]}}^{2}+{[{\rm{F16BP}}]}^{2}}.$$

where ${V}_{{{\rm{\max }}}_{[{\rm{G}}3{\rm{P}}]}}$ is the limiting value of the reaction rate of G3P, ${K}_{{m}_{[{\rm{G3P}}]}}$ is the substrate concentration that gives half the maximal rate, $[{\rm{G}}3{\rm{P}}]$ is the concentration of G3P, and *q* is the portion of F16BP that is used in the production of G3P.

Down the lactate producing pathway in glycolysis, we move from modeling the reaction resulting in F16BP to the next committed step, the production of pyruvate. To simplify things, we take F16BP as a substrate and pyruvate kinase denoted by PK as the enzyme in the production of pyruvate, PYR, such that

$$[{\rm{F}}16{\rm{BP}}]+[{\rm{PK}}]\leftrightharpoons [{\rm{F}}16\mathrm{BP}/\mathrm{PK}]\to [{\rm{PYR}}]+[{\rm{PK}}].$$

The reaction rate of pyruvate in the model is governed by

$${\nu }_{[{\rm{PYR}}]}=\frac{(1-q)\,{V}_{{{\rm{\max }}}_{[{\rm{PYR}}]}}{[{\rm{F}}16{\rm{BP}}]}^{2}}{{K}_{{m}_{[{\rm{PYR}}]}}^{2}+{[{\rm{F}}16{\rm{BP}}]}^{2}}$$

where ${V}_{{{\rm{\max }}}_{[{\rm{PYR}}]}}$ is the limiting value of the reaction rate of PYR, ${K}_{{m}_{[{\rm{PYR}}]}}$ is the substrate concentration that gives half the maximal rate, and $(1\,-\,q)$ is the portion of F16BP that eventually leads to the production of PYR.

Finally the last step of aerobic glycolysis is the production of lactate, LACT, where lactate dehydrogenase (LDH) is the enzyme and PYR the substrate^21^:

$$[{\rm{PYR}}]+[{\rm{LDH}}]\leftrightharpoons [\mathrm{PYR}/\mathrm{LDH}]\leftrightharpoons [{\rm{LACT}}]+[{\rm{LDH}}].$$

The reaction rate of lactate is governed by

$${\nu }_{[{\rm{LACT}}]}=\frac{(1-\rho )\,{V}_{{{\rm{\max }}}_{[\mathrm{LACT}]}}[{\rm{PYR}}]}{{K}_{{m}_{[{\rm{LACT}}]}}+[{\rm{PYR}}]}.$$

where $[{\rm{LACT}}]$ is the concentration of lactate, $\rho$ is the percent of ROS produced by leakage of the mitochondrial respiratory chain, ${V}_{{{\rm{\max }}}_{[{\rm{LACT}}]}}$ is the limiting value of the reaction rate of LACT, and ${K}_{{m}_{[{\rm{LACT}}]}}$ is the substrate concentration that gives half the maximal rate.

During aerobic glycolysis, the 6-carbon atoms of glucose are not transformed entirely into lactate. Some are diverted to DHAP to produce triglycerides that are finally incorporated into phospholipids of the cone OS. There is no oxidative stress resulting from the production of lactate. Lactate dehydrogenase produces lactate from pyruvate by transferring the electron from the reduced form of nicotinamide adenine dinucleotide (NADH) to the oxidized form of nicotinamide adenine dinucleotide (NAD+) which is reused upstream in the glycolysis pathway. The conversion of pyruvate to lactate is reversible and the direction of the reaction depends on lactate dehydrogenase subtypes. Photoreceptors express the subtype A which favors the production of lactate from pyruvate and the RPE expresses the subtype B which favors the production of pyruvate from lactate^22^. This is necessary for the astrocyte neuronal lactate shuttle (ANLS). Nevertheless, lactate does not protect cone photoreceptors *in vitro*^10^ and retinal ANLS is debated^23^.

In the healthy retina, ROS are produced from glucose through glycolysis and mitochondrial OXPHO as well as from photo-oxidation^9^. In a rod-less retina, the cones are under hyperoxia due to the fact that choroid circulation is not regulated by the reduction of photoreceptors^15,24^. There is a partitioning in the cone between glucose going through aerobic glycolysis (for cone OS renewal) and glucose going through oxidative phosphorylation (main ATP production step). RdCVF stimulates glucose through aerobic glycolysis, while ROS favors glucose via CO_2_ + NADPH and inhibits both glucose via aerobic glycolysis and glucose through oxidative phosphorylation. In the PPP, we mathematically consider the production of NADPH described in equation (2), and the inhibition of ROS, illustrated by the second term in the $\frac{d[{\rm{ROS}}]}{dt}$ equation in Section 2.2 below. ROS are inhibited by rod-derived cone viability factor long (RdCVFL) in its reduced form, through the production of NADPH via the PPP^14^. RdCVFL is expressed by the rods and the cones. RdCVF is a splicing variant of the nucleoredoxin-like-1 (*NXNL1*) gene corresponding to intron retention and a conserved in-frame stop codon. Splicing of that unique intron produces a messenger ribonucleic acid (mRNA) encoding an active thioredoxin enzyme, RdCVFL, L for the extension in the C-terminal part of the protein^25^. ROS is generated from glucose, through glycolysis and pyruvate which is transferred to the mitochondria by the mitochondrial pyruvate carrier (MPC). We model the rate of production of ROS with respect to time as

3 $${\nu }_{[{\rm{ROS}}]}=\frac{\rho [{\rm{PYR}}]}{1+\omega [{\rm{NADPH}}]}+r$$

where $\rho$ is the percentage of ROS produced by leakage of the mitochondrial respiratory chain while $\omega$ measures the amount of ROS detoxified/reduced in this process. The parameter *r* is the contribution to ROS by photo-oxidation and other damaging mechanisms.

### Molecular Dynamics

The following set of differential equations describes the time-dependent behavior of the system at the molecular level. For [g], [G6P], [F16BP], and [PYR], the change in the concentration of substrate with respect to time is equal to the reaction rate for that concentration of substrate minus the reaction rate of the concentration of product resulting from catabolizing that substrate with an enzyme. For [NADPH] and [LACT], the change in the concentration of substrate with respect to time is equal to the reaction rate of the substrate concentration minus the loss of substrate concentration due to natural processes that oxidize or degrade these molecules. The loss of substrate concentration in this instance is represented by $\tau$ and $\psi$, where $\tau$ is the rate at which [NADPH] is lost and $\psi$ is the rate at which [LACT] is lost. The rate of change of [G3P] is the difference between the reaction rate for [G3P] and the amount of [G3P] removed for the renewal of cone OS due to phospholipid synthesis. The latter process is described by $\eta$, the fraction of G3P removed for phospholipid synthesis and *α*, the utilization of G3P by a single cone for OS production. The rate of change of [ROS] with respect to time is equal to the reaction rate of [ROS] minus the reduction of [ROS] due to RdCVFL, where *δ*_*L*_ is the rate at which ROS is reduced due to RdCVFL.

$$\begin{array}{rcl}\frac{d[{\rm{g}}]}{dt} & = & {\nu }_{[{\rm{g}}]}-{\nu }_{[{\rm{G}}6{\rm{P}}]}\\ \frac{d[{\rm{G}}6{\rm{P}}]}{dt} & = & {\nu }_{[{\rm{G}}6{\rm{P}}]}-{\nu }_{[{\rm{F}}16{\rm{BP}}]}-{\nu }_{[{\rm{NADPH}}]}\\ \frac{d[{\rm{F}}16{\rm{BP}}]}{dt} & = & {\nu }_{[{\rm{F}}16{\rm{BP}}]}-{\nu }_{[{\rm{PYR}}]}-{\nu }_{[{\rm{G}}3{\rm{P}}]}\\ \frac{d[{\rm{G}}3{\rm{P}}]}{dt} & = & {\nu }_{[{\rm{G}}3{\rm{P}}]}-\alpha \eta C[{\rm{G}}3{\rm{P}}]\\ \frac{d[{\rm{NADPH}}]}{dt} & = & {\nu }_{[{\rm{NADPH}}]}-\tau [{\rm{NADPH}}]\\ \frac{d[{\rm{PYR}}]}{dt} & = & {\nu }_{[{\rm{PYR}}]}-{\nu }_{[{\rm{LACT}}]}\\ \frac{d[{\rm{LACT}}]}{dt} & = & {\nu }_{[{\rm{LACT}}]}-\psi [{\rm{LACT}}]\\ \frac{d[{\rm{ROS}}]}{dt} & = & {\nu }_{[{\rm{ROS}}]}-{\delta }_{L}[{\rm{ROS}}]\end{array}$$

### Cellular Dynamics

Following the models in^5,7,11–13^, the new equation describing the change in *C* with respect to time is

$$\frac{dC}{dt}=CT{a}_{c}([{\rm{G}}3{\rm{P}}])-C{\mu }_{c}-C{\mu }_{[{\rm{ROS}}]}[{\rm{ROS}}],$$

where *C* represents the sum of the proportion of full length of each cone OS in the retina, *μ*_*c*_ is the metabolism associated with OS shedding of *C*, and *μ*_[ROS]_ is the rate of cone degeneration due to ROS. The cellular metabolism in cones associated with their OS renewal is described by

4 $${a}_{c}([{\rm{G}}3{\rm{P}}])=\epsilon \alpha [{\rm{G}}3{\rm{P}}]$$

where *α* is the utilization of G3P for phospholipid synthesis. The parameter $\epsilon$ is the conversion factor of G3P into cone OS discs in the renewal process.

We define the proportion of full length of an OS to be its current length divided by the maximum OS length. The proportion of full length of each OS fluctuates throughout the day due to periodic shedding and continuous renewal such that at any time it can take any value between 0 and 1. In a healthy retina this value would be far away from zero. Thus, we let *C* represent the sum of the proportion of full length of each cone OS in the retina. Similarly, we let *R*_*n*_ represent the sum of the proportion of full length of each rod OS in the retina. Initially, we assume that some portion of OS are not at full length because of periodic shedding. We let *T* represent the retinal pigment epithelium (RPE) supplied neuroprotective factors, growth factors, and nutrients. We refer to *T* as the trophic pool mediated by the RPE.

The equations modeling the sum of the proportion of full length of each rod OS in the retina, *R*_*n*_, and the tropic pool mediated by the RPE, *T*, take the form

$$\begin{array}{rcl}\frac{d{R}_{n}}{dt} & = & {R}_{n}T{a}_{n}-{R}_{n}{\mu }_{n},\\ \frac{dT}{dt} & = & T({\rm{\Gamma }}-kT-{\beta }_{n}{R}_{n}-\gamma C),\end{array}$$

where *μ*_*n*_ is the metabolism associated with OS shedding of *R*_*n*_, ${\rm{\Gamma }}$ is the total inflow rate into the trophic pool, $\kappa$ is the limiting capacity of trophic factors, *β*_*n*_ is the removal of nutrients from *T* by *R*_*n*_, *γ* is the removal of nutrients from *T* by *C*, *a*_*n*_ is the metabolism associated with OS renewal of *R*_*n*_.

### Simulation Results

**Figure 3:**
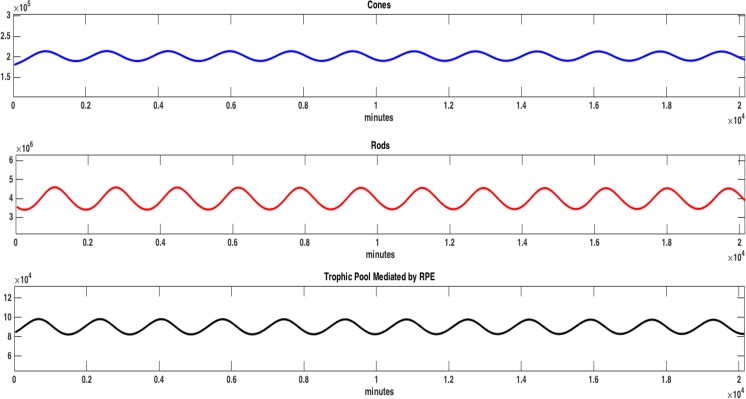
Plot of *C*, *R*_*n*_, and *T* vs. time (in minutes) when all processes are functioning properly for 20,160 minutes (14 days). The parameters used for the simulation are those listed in Table 2. The ratio of rods to cones is about 20:1, with *C*(20,160) = 1.93 × 10^5^.

**Figure 4:**
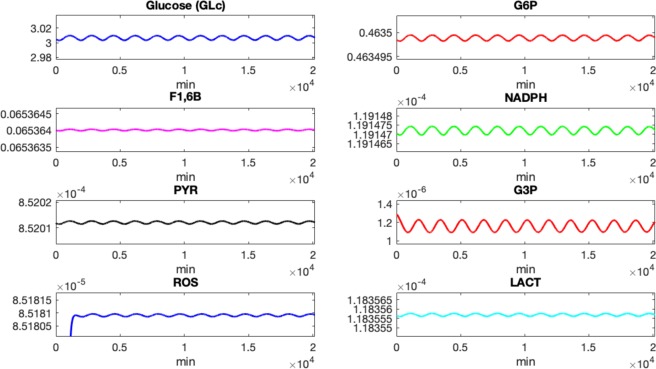
Plot of concentrations of various biochemical quantities (in mM) vs. time (in minutes) that arise in aerobic glycolysis, PPP, and OXPHO. The parameters used for the simulation are those listed in Table 2. Simulations are from time $t=0$ minutes to *t* = 20,160 minutes (which is 14 days).

In our analysis, we consider four cases, the latter two involving RdCVF: (i) the first case is when all process and mechanisms, defined by the parameters, are functioning properly; (ii) another is when the cones do not efficiently utilize the glucose that enters the cell for phospholipid synthesis, i.e., OS renewal (illustrated by a small $\epsilon$ value in (4)); (iii) a third case is one in which the rods are not synthesizing enough RdCVF needed in a healthy retina (illustrated by a small *δ* value in (5)); and (iv) the final case is where there is no RdCVF (*δ* = 0); these results are summarized in Table 1. In order to isolate the specific mechanisms driving these cases, the initial amounts of *C*, *R*_*n*_, and *T* as well as the various parameter values were maintained at the same values in all four cases; see Table 2. Simulations of our mathematical model show that qualitatively the behavior of the system is as expected with oscillations both at the cell population and molecular level. See Figs 3 and 4 where the long term dynamics are plotted.

**Table 1:** Summary of sensitivity analysis results.

Case	Sensitivity Highlights	Values of *C*	Key Processes
**Case 1**: All Processes Functioning Properly	• *C* is not sensitive to RdCVF	• *C*(*t*_1_) = 1.8 × 10^5^	• Aerobic glycolysis
	• *C* is sensitive to ${K}_{{m}_{i}}$, and ${V}_{{{\rm{\max }}}_{i}}$ for *i* = [PYR], [LACT] at all time points considered	• *C*(*t*_4_) = 1.9 × 10^5^	• Cone OS renewal
	• *C* is sensitive to $\eta$, *q*, ${K}_{{m}_{[{\rm{G}}3{\rm{P}}]}}$, and ${V}_{{{\rm{\max }}}_{[{\rm{G}}3{\rm{P}}]}}$ at all time points considered	• The ratio of rods to cones is approximately 20:1 all time points considered	• Competition for resources
	• *C* is sensitive to various cell population level parameters at all time points considered		
**Case 2**: Insufficient RdCVF *δ* = 65 × 10^−8^	• At *t*_2_, *t*_3_, and *t*_4_, *C* is sensitive to RdCVF (this is the only case where sensitivity to RdCVF occurs)	• *C*(*t*_1_) = 1.6 × 10^5^	• Cone OS renewal
	• *C* is not sensitive to ${K}_{{m}_{i}}$ and ${V}_{{{\rm{\max }}}_{i}}$ for *i* = [PYR], [LACT] but it is to [G6P]_0_, ${K}_{{m}_{[{\rm{F}}1{\rm{B}}6{\rm{P}}]}}$, ${V}_{{{\rm{\max }}}_{[{\rm{F}}1{\rm{B}}6{\rm{P}}]}}$, and [F16BP]_0_ at *t*_1_	• *C*(*t*_4_) = 3.6 × 10^3^	• Competition for resources
	• *C* is also sensitive to $\eta$, *q*, ${K}_{{m}_{[{\rm{G}}3{\rm{P}}]}}$, and ${V}_{{{\rm{\max }}}_{[{\rm{G}}3{\rm{P}}]}}$ at *t*_1_		Note: At equilibrium, glucose is not entering the cell near its maximum rate indicated by
	• *C* is sensitive to various cell population level parameters at all time points considered		$\frac{{V}_{{{\rm{\max }}}_{[{\rm{g}}]}}{(\delta {R}_{n})}^{2}}{{K}_{{m}_{[{\rm{g}}]}}^{2}+{(\delta {R}_{n})}^{2}}\ll {V}_{{{\rm{\max }}}_{[{\rm{g}}]}}$
**Case 3**: No RdCVF *δ* = 0	• *C* is not sensitive to RdCVF	• *C*(*t*_1_) = 1.6 × 10^5^	• Cone OS renewal
	• *C* is not sensitive to ${K}_{{m}_{i}}$ and ${V}_{{{\rm{\max }}}_{i}}$ for *i* = [PYR], [LACT]	• *C*(*t*_4_) = 1	• Competition for resources
	• At *t*_1_ and *t*_2_, *C* is sensitive to [F1B6P]_0_, $\eta$, *q*, ${K}_{{m}_{[{\rm{G}}3{\rm{P}}]}}$, and ${V}_{{{\rm{\max }}}_{[{\rm{G}}3{\rm{P}}]}}$		
	• At *t*_1_, *C* is sensitive to *α* (this is the only case where sensitivity to *α* occurs)		
	• *C* is sensitive to various cell population level parameters at all time points considered		
**Case 4**: Inefficient use of glucose for cone OS renewal $\epsilon =9.99\times {10}^{-4}$	• The sensitivity highlights are the same as those in case one	• *C*(*t*_1_) = 1.5 × 10^5^	• Aerobic glycolysis
		• *C*(*t*_4_) = 5.7 × 10^3^	• Cone OS renewal
		• *C*(*t*_4_) is smaller in this case compared to case one	• Competition for resources

We used uncertainty and sensitivity analysis (see Section 4.2) to analyze the sensitivity of *C* (defined in Section 2.3) with respect to the model parameters at four time points (*t*_1_ = 60 min, *t*_2_ = 1 day, *t*_3_ = 7 days, and *t*_4_ = 14 days) to verify the cones’ reliance on rods via RdCVF and to gain insight into an understanding of cone vitality. We considered four cases, two involving RdCVF, and concluded that cone OS production is driven by combinations of three key sets of processes. The first set of processes included sensitivity to [LACT] and [PYR] parameters which indicated a reliance on aerobic glycolysis for cone OS production; see Equations 10 and 11. The second set of processes involved sensitivity to [G3P] parameters; see Equation 8. In this instance, we concluded that cone OS production is supported by cone OS renewal. Finally, the third set of processes involved a reliance on cell population level parameters allowing us to conclude that competition for resources affects cone OS production; see Equations 13–15. When the rods are degenerating, leading to low/insufficient RdCVF (*δ*) synthesis, trophic factors become crucial in maintaining *C*. It was in this case, insufficient RdCVF, that we saw *C* sensitive to RdCVF indicating a reliance of cones on rods via RdCVF. In the absence of RdCVF (*δ* = 0), glucose uptake diminishes significantly and cone OS production cannot be maintained. All parameters are defined in Sections 2.1–2.3. Parameter values are in Table 2. PRCC values for each case are included in Supplemental Information A.6.

**Table 2:** Table of parameters.

Parameter	Value	Source
[*G*]	5 mM	*
*λ*	0.0775 mM^−1^	+
${V}_{{{\rm{\max }}}_{[{\rm{g}}]}}$	1.2 mM · min^−1^	^38^
${K}_{{m}_{[{\rm{g}}]}}$	19 mM	^38^
*δ*	65 × 10^−6^ mM	*
${V}_{{{\rm{\max }}}_{[{\rm{G}}6{\rm{P}}]}}$	0.1845 mM · min^−1^	^39^
${K}_{{m}_{[{\rm{G}}6{\rm{P}}]}}$	0.09 mM	^38^^,^*
${V}_{{{\rm{\max }}}_{[{\rm{F}}16{\rm{BP}}]}}$	1.365 mM · min^−1^	^40^
${K}_{{m}_{[{\rm{F}}16{\rm{BP}}]}}$	3.5 mM	^40^
[PEP]_*eq*_	6.375 × 10^−4^ mM	*
${V}_{{{\rm{\max }}}_{[{\rm{G}}3{\rm{P}}]}}$	0.15 mM · min^−1^	*
${K}_{{m}_{[{\rm{G}}3{\rm{P}}]}}$	0.143 mM	*
*q*	0.18	+
${V}_{{{\rm{\max }}}_{[{\rm{NADPH}}]}}$	0.228 mM · min^−1^	^41^
${K}_{{m}_{[{\rm{NADPH}}]}}$	0.45 mM	^41^
$\tau$	1.5 × 10^3^ min^−1^	+
${V}_{{{\rm{\max }}}_{[{\rm{PYR}}]}}$	0.3915 mM · min^−1^	^39^
${K}_{{m}_{[{\rm{PYR}}]}}$	1.7 mM	^39^
${V}_{{\rm{\max }}{}_{[{\rm{LACT}}]}}$	0.14 mM · min^−1^	+
${K}_{{m}_{[{\rm{LACT}}]}}$	0.125 mM	^42^
${\rm{\Psi }}$	8 min^−1^	+
$\eta$	2 × 10^−2^	+
$\rho$	0.001	*
$\omega$	2 mM^−1^	+
*r*	0 mM · min^−1^	+
*δ*_*L*_	0.01 min^−1^	+
*α*	1 min^−1^	+
*μ*_*c*_	3.5 × 10^−3^ min^−1^	^12^
*μ*_[ROS]_	6.94 × 10^−5^ mM^−1^ · min^−1^	+
*a*_*n*_	6.94 × 10^−8^ min^−1^	^12^
*μ*_*n*_	6.25 × 10^−3^ min^−1^	^12^
${\rm{\Gamma }}$	2.22 × 10^−3^ min^−1^	^12^
$\kappa$	3.47 × 10^−11^ min^−1^	^12^
*β*_*n*_	5.56 × 10^−10^ min^−1^	^12^
*γ*	6.94 × 10^−11^ min^−1^	^12^
$\epsilon$	3.33 × 10^−2^ mM^−1^	+
*p*	1 × 10^−15^ mM · min^−1^	+
[g]_0_	3 mM	^43^
[G6P]_0_	0.45 mM	+
[F16BP]_0_	0.06 mM	+
[G3P]_0_	2.5 × 10^−6^ mM	+
[NADPH]_0_	1.2 × 10^−4^ mM	+
[PYR]_0_	7.5 × 10^−4^ mM	+
[LACT]_0_	1 × 10^−4^ mM	+
[ROS]_0_	0 mM	+
*C*_0_	0.18 × 10^6^	^7^
${R}_{{n}_{0}}$	3.6 × 10^6^	^7^
*T*_0_	8.4 × 10^4^	^12^

These are parameters when all processes in the retina are functioning properly. The parameters with a subscript zero represent the initial values for the biochemical concentrations and cell population level variables.

^+^These values were estimated for simulation analysis.

*The parameters were taken from a model of cone-enriched culture system that was used originally to identify RdCVF by high content screening^6^; see Supplemental Material A.2.

In Fig. 3, the outputs for *C* qualitatively represent the sum of the proportion of full length of each cone OS, and the outputs for *R*_*n*_ qualitatively represent the sum of the proportion of full length of each rod OS. In the long run, the peaks and valleys of the oscillations in Fig. 3 reach the same maximum and minimum value, respectively, which implies that the number of OS is staying approximately constant over time. Thus, the rods and cones are not dying (as expected when all processes are functioning properly). In Fig. 3 we also see that the trophic pool, which is mediated by the RPE, oscillates. The trophic pool contains nutrients necessary for cone and rod survival. Thus, the level of the trophic pool should increase as nutrients are replenished and decrease as the rods and cones take nutrients. The interactions at the cellular level have been extensively studied using mathematical models in^12,13^. Following the assumptions of these models, the rods and the cones take more nutrients from the trophic pool than they contribute (as recycled products), and the production of the trophic factors is logistic in nature. Thus, the trophic pool, with its carrying capacity of ${\rm{\Gamma }}$/$\kappa$, will reach an equilibrium in the absence of photoreceptors instead of growing without bound, which we confirmed with simulation and *in silico* experiments. Biological experiments suggest that the ratio of rods to cones should be approximately 20:1 and the model simulations agree with this when all processes are functioning properly. (See Supplemental Information A.4).

Simulations for the molecular outputs are presented in Fig. 4. Here the concentrations of biochemical quantities (in mM) with respect to time (in minutes) are displayed. These quantities are the outputs of the chemical reactions mainly involved in the committed steps of the aerobic glycolysis process within a single cone cell; see Fig. 1. After a very short period of transience, the solutions approach the inherent oscillatory behavior observed in the real system. Thus oscillations can be interpreted as being consistent with circadian rhythms of photoreceptor OS phagocytosis/shedding and expression of *NXNL1*^26,27^. Very few experiments have begun to isolate the effect of circadian rhythm on specific components of the system although there is evidence in some of circadian rhythm (where oscillations persist even under constant dark conditions).

**Figure 5:**
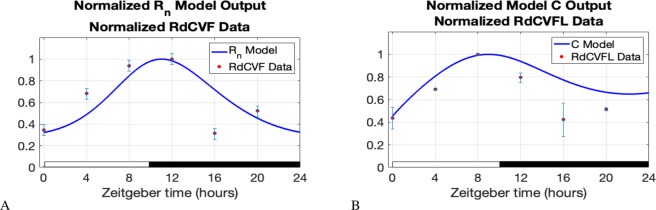
We normalized RdCVF expression data by dividing by the largest RdCVF value. In a similar manner, we normalized the RdCVFL expression data. We also normalized the model output values for *C* and *R*_*n*_ by dividing by the model’s maximum *C* and *R*_*n*_ values, respectively, on the time interval from 0 minutes to 1,440 minutes (24 hours). We let *R*_*n*_(0) = 2,088,000 and *C*(0) = 7,380 in the model so that the initial normalized data and normalized model output values are the same. All other parameter values are the same as they appear in Table 2. RdCVF and RdCVFL expression data was collected at 0, 4, 8, 12, 16, and 20 hours on Zeitgeber time; see Fig. 10 in Supplemental Material A.3.

To demonstrate the presence of a circadian rhythm in our model compared with experimental data, we utilize RdCVF and RdCVFL expression data from our experiments; see Fig. 5 below and Fig. 10 in Supplemental Material A.3. RdCVF and RdCVFL expression data points were recorded at times 0, 4, 8, 12, 16, and 20 hours on Zeitgeber time. Neural retina of wild-type mice were dissected and the ribonucleic acid (RNA) was purified using cesium chloride (CsCl) ultracentrifugation^28^. 500 ng of RNA were used for complementary deoxyribonucleic acid (cDNA) reverse transcription (Superscript III enzyme) with random hexamer, and quantitative reverse transcription polymerase chain reaction (RT-PCR) was performed; see Supplemental Material A.3. RdCVF is expressed exclusively by the rods whereas RdCVFL is expressed by both the rods and the cones. The expression of RdCVF and RdCVFL is gradually increased after light onset which follows the phagocytosis of photoreceptor OS^29^. Interestingly, increased RdCVF expression by rods corresponds to the need to synthesize triglycerides from glucose through the Kennedy pathway to renew cone OS^9^.

When all process are functioning properly, we anticipate oscillations in order for the rods and cones to be thriving. The rods are synthesizing RdCVF, which binds to the BSG1/GLUT1 complex accelerating the entry of glucose into the cone cell, and thereby stimulating aerobic glycolysis. As glucose enters the cell triggering aerobic glycolysis, glucose may either travel down the lactate producing pathway, the Kennedy pathway in the direction of phospholipids synthesis, or be diverted to the PPP for the reduction of ROS, illustrated in Fig. 1. During the aerobic glycolysis process, the product from a previous reaction is used as the substrate for the following reaction. Thus the concentrations are expected to oscillate in coordination.

**Figure 6:**
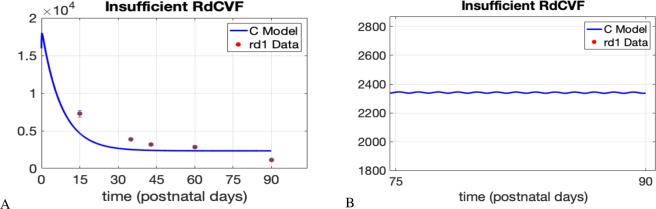
Model validation with *rd1* cone density data. The cone density data represents cone cell count per mm^2^. In^30^, each square millimeter is referred to as a field. There are approximately 300 fields with surface area 0.0376 mm^2^. Our mathematical model produces values for *C* throughout the retina instead of within a particular surface area. Thus, we divide the model output, *C*, by (300 · 0.0376). The data points in the plots correspond to the mean cone densities at postnatal days 15, 35, 43, 60, and 90. In the model simulation we consider a time span of *t*_0_ = 0 minutes to *t*_*f*_ = 12.96 × 10^4^ minutes (90 days), *δ* = 30 × 10^−9^ and *μ*_*c*_ = 0.09 × 10^−3^. All other parameter values are the same as in Table 2. In (**B**), the image is zoomed in to show the presence of small oscillations even around the 90 day time point.

As further validation of our model, we compare the output for *C* with the series 1 *rd1* global automated data found in Additional File 3 of ^30^. The data represents the cone density in the *rd1* mouse using the global automated method for cone quantification. The *rd1* mouse model is one in which recessive RP is present and thus the rods die due to a mutation in the rod photoreceptors. This data shows that the loss of rods and RdCVF leads to cone degeneration and renewal/shedding of cone OS is disrupted. In our mathematical model this translates to reducing *δ*, the RdCVF synthesized by rods, as well as *μ*_*c*_, the metabolism associated with OS shedding of cones, while keeping all other parameter values the same as in Table 2. Simulating our model for 90 days we see an exponential decay in *C* comparable to the death kinetics of cones in the *rd1* data; see Fig. 6 below. For small *δ* and *μ*_*c*_ values the model decreases rapidly and then settles down to a very small *C*-value or zero for extremely small *δ* values. In Fig. 6 the *C*-value decreases rapidly for the first 30 days, due to lack of RdCVF. In the model output, the disruption in the metabolism associated with OS shedding is observed through the small amplitude in the oscillations of *C*; see panel B in Fig. 6.

### Uncertainty and Sensitivity Analyses

In the work presented here, we focus on understanding the driving mechanisms involved in cone metabolism and degeneration. Uncertainty analysis (UA) allows us to determine the uncertainty in analytical or numerical results that comes from uncertainty in input parameters^31,32^. Sensitivity analysis (SA) quantifies the contributions of individual inputs to model outputs, indicating the parameters that have the most significant affect on the output variables. We limit our sensitivity analysis to the effect on the cones by changes in the parameter inputs. We performed UA and SA in MATLAB using Latin Hypercube Sampling (LHS) followed by partial rank correlation coefficient (PRCC) analysis; see Section 4.2. This allowed us to conduct a global sensitivity analysis of *C*. We conducted these analyses using 500 runs, meaning that the parameter sample space for each parameter in the mathematical model was partitioned into 500 equiprobable intervals that were sampled to create the LHS matrix. We ran the simulations for each case (discussed in Section 2.4) using ${t}_{0}=0$ and ${t}_{f}=2,000$, and then used the final output values of the simulations as the initial conditions in the code for the UA and SA. We did this to remove any effect of the initial transients in the computation of the PRCC values.

Results are presented in two different forms in this section. One is a PRCC bar graph that gives the sensitivity of the output, *C*, to changes in the parameters. The PRCC bar graphs contain only the parameters with significant PRCC values at a specific time point; see Section 4.2. The second form is the flow chart of our molecular model with the significant mechanisms defined by the parameters and given by the PRCC plots superimposed on it. These results are presented at $t=60\,{\rm{\min }}$, 1 day, 7 days, and 14 days; see Table 1.

**Figure 7:**
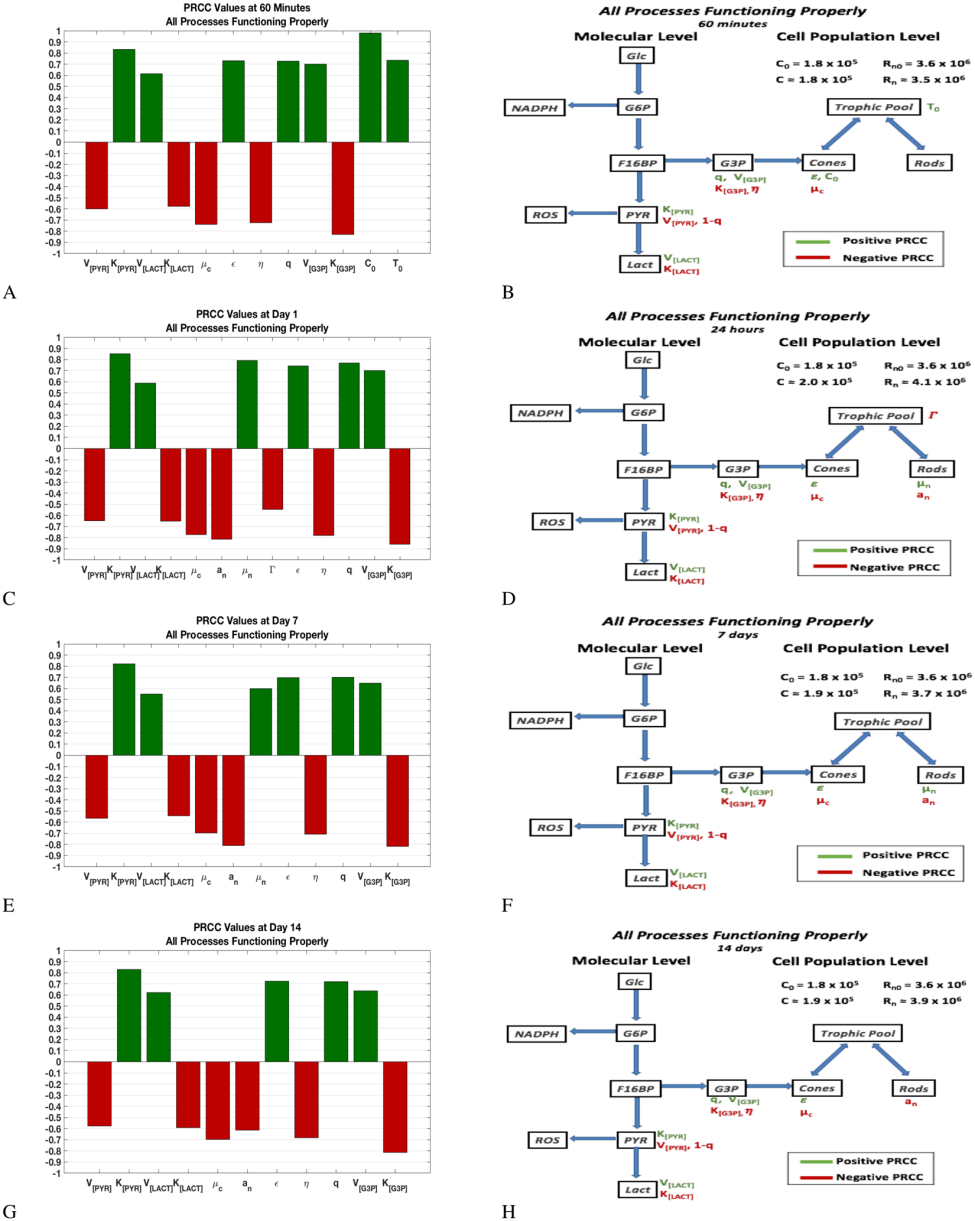
PRCC plots and corresponding effects for a retina in which all processes are functioning properly. The left panels give the partial rank correlation coefficient (PRCC) plots for parameters with significant PRCC values while the right panels give the location of those parameters in the flow diagram. The value of *C* and *R*_*n*_ at the given time snapshot are given in the right panels. The PRCC values are given in Table 15 (in Supplemental Information). See Sections 4.2 and 2.5 for details on PRCC analysis.

When all the processes are functioning properly (i.e., in a healthy retina), the analysis reveals that cone OS production is driven by three key sets of processes, two at the molecular level within the cones and the third at the population (of cells) level; see Fig. 7. The first is the contribution of F16BP to DHAP and GAP that ensure the reactions down the glycolysis pathway; see Fig. 1. The second set of processes involve the availability of G3P and the efficiency to utilize G3P to make phospholipids. The third set involves the competition of cones and rods for resources. These processes are highlighted in panels B, D, F, and H of Fig. 7 in green (for changes in processes that result in an increase in *C* when everything is held fixed except for the particular parameter/process in question) and red (for changes in processes that lead to a decrease in *C* when everything is held fixed except for the particular parameter/process in question). At day 14, the three sets of key processes remain but the macroscopic influence of the rod cells is only in regards to their rate of energy uptake and metabolism associated with their OS renewal since enough trophic factors are assumed to be available for a healthy retina. The analysis reveals that any desire to increase OS disc production of the cones when the system is operating at or near healthy levels would require focusing on one of these three key sets of processes mentioned above.

In the first set of processes of this case, for all time snapshots considered, lesser amounts of F16BP inciting PYR and LACT production, leads to better cone OS renewal. This is reflected in the sensitivity of the cone OS to the parameters *V*_max_ and *K*_*m*_ involved in the production of PYR and LACT. Changes in these processes (defined by *V*_max_ and *K*_*m*_) that lead to a larger production of PYR and slower catalytic reaction to LACT result in less cone OS renewal. In our model the relation of [PEP]_*eq*_ to [PYR] determines if PEP and PYR accumulate and if glucose is rerouted into the PPP and interrupts the catalytic reaction of G3P and production of phospholipids; see Fig. 2, right panels in Fig. 7, and Supplemental Information Table 15.

The second set of key processes is responsible for the availability of G3P and the efficiency to renew OS discs. The latter is quantified by, $\epsilon$, the conversion factor of G3P into OS disc renewal. The former is quantified by parameters involved in the production of G3P, specifically *q*, ${V}_{{{\rm{\max }}}_{[{\rm{G}}3{\rm{P}}]}}$, ${K}_{{m}_{[{\rm{G}}3{\rm{P}}]}}$, and the fraction of G3P removed for phospholipid synthesis, $\eta$; see Fig. 7. An increase in ${V}_{{{\rm{\max }}}_{[{\rm{G}}3{\rm{P}}]}}$ and *q*, the proportion of F16BP leading to an increase in [G3P], (as illustrated in Fig. 1) as well as a decrease in ${K}_{{m}_{[{\rm{G}}3{\rm{P}}]}}$ and $\eta$ results in more [G3P] that can be utilized for phospholipid synthesis and OS renewal. All these effects involving the second set of processes are observed for all times considered.

The third set of key processes ties into the trophic factors available to both cones and rods as well as their metabolism associated with shedding and renewal at the population (of cells) level. Reduced availability of trophic factors (due to greater use by the rods) can slow production of the cone OS discs. At 60 minutes, increases in the initial amount of trophic pool mediated by the RPE, *T*_0_, and the *C*, *C*_0_, lead to more cone OS; an increase in the cone metabolism associated with shedding, *μ*_*c*_, results in a reduction of cone OS, as one would expect. By 24 hours, the cone OS are no longer sensitive to changes in *T*_0_ and *C*_0_. However, an increase in the total inflow rate of trophic factors, ${\rm{\Gamma }}$, and metabolism associated with rod OS renewal, *a*_*n*_, favor the rods and negatively affect cones. Changes in the metabolism associated with shedding of cones, *μ*_*c*_, and of rods, *μ*_*n*_, have opposite effects in the cone OS with the negative effect of *μ*_*c*_ persisting; see Fig. 7 and Supplementary Information Table 15. At 7 days, the sensitivity of cone OS to the metabolism associated with shedding of both cones and rods and that of rod renewal remain with the effect of ${\rm{\Gamma }}$ gone. By day 14, the effect of changes in *μ*_*n*_ on *C* disappears.

When glucose is used inefficiently (illustrated by an extremely small $\epsilon$), our analysis demonstrates that the influence on *C* comes from the same three key sets of processes as in the healthy retina case. The strength of their respective contributions, as measured by the PRCC values, is approximately the same as in the healthy case. The results are given in Fig. 12 and Table 16 (both in Supplemental Information). However, there is a rapid decrease in *C* due to this inefficient use of glucose. Decreasing $\epsilon$ to 3% of its regular value, *C* decreased significantly from $1.5\times {10}^{5}$ at time $t=60\,{\rm{minutes}}$ to $5.7\times {10}^{3}$ at time $t=14\,{\rm{days}}$.

**Figure 8:**
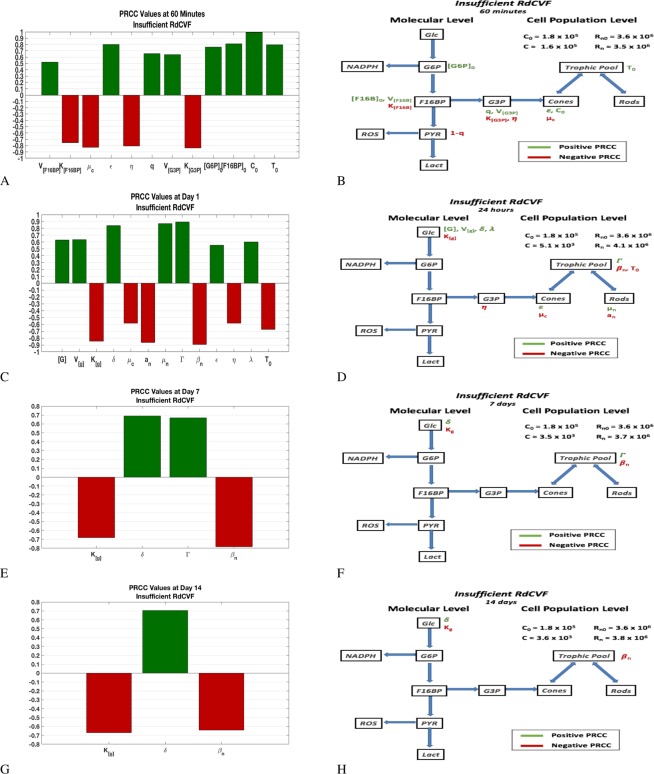
PRCC plots and corresponding effect on flow diagram for low levels of RdCVF. The two top panels, (A and B) are for 1 hour, the next two panels, (B and D), for 1 day, the next two panels, (E and F), for 7 days, and the two bottom panels, (G and H), are for 14 days. The left panels give the partial rank correlation coefficient (PRCC) plots for parameters with significant PRCC values while the right panels give the location of parameters with significant PRCC values in the flow diagram. The value of *C* is significantly lower compared to the healthy eye but levels off to a reduced value. The value of *C* and *R*_*n*_ at every time snapshot is given in each of the figures in the right panel. The PRCC values are given in Supplemental Information Table 17. See Sections 4.2 and 2.5 for details on PRCC analysis.

The drastic reduction but not elimination of RdCVF fundamentally affects the system in a different way because the amount of F16BP available is altered. Two of the three processes, efficiency of making phospholipids and competition by the rods for resources remain. However, instead of the influence by PYR (because of its effect on the glycolysis pathway vs. PPP), we see that it is the intracellular level of glucose (before the G6P reaction and potential PPP selection) that significantly affects the system. In this case, the limiting value of the chemical reaction ${\nu }_{[{\rm{g}}]}=\frac{{V}_{{{\rm{\max }}}_{[{\rm{g}}]}}{(\delta {R}_{n})}^{2}}{{K}_{{m}_{[{\rm{g}}]}}^{2}+{(\delta {R}_{n})}^{2}}$ is much less than ${V}_{{{\rm{\max }}}_{[{\rm{g}}]}}$ in the long run, indicating that at equilibrium glucose is not entering the cell near its maximum rate. Biologically, the depletion or lack of RdCVF has only been associated with degenerating rods or rod-less retinas, respectively, such as the case of RP^6,8,9,33,34^. Thus in this disease-free mathematical model, it is safe to associate the depletion of RdCVF with rod degeneration (rather than other potential factors that might affect RdCVF but are not currently known or identified). If the rods are dying, then there is further reduction of RdCVF as time goes on, which results in a magnification of the decrease of glucose entering the cone cell to be metabolized via aerobic glycolysis. Interestingly, the significance of $\epsilon$, which represents the efficiency of the cones to use G3P to make phospholipids, decreases over time and it is the overall RdCVF levels, *δ*, and the effectiveness of the rods utilizing trophic factors, *β*_*n*_, that have the most significant impact. Thus, making sure sufficient levels of RdCVF and trophic factors are available will be the best strategies to increase cone OS renewal; see Fig. 8 and Supplementary Information Table 17.

**Figure 9:**
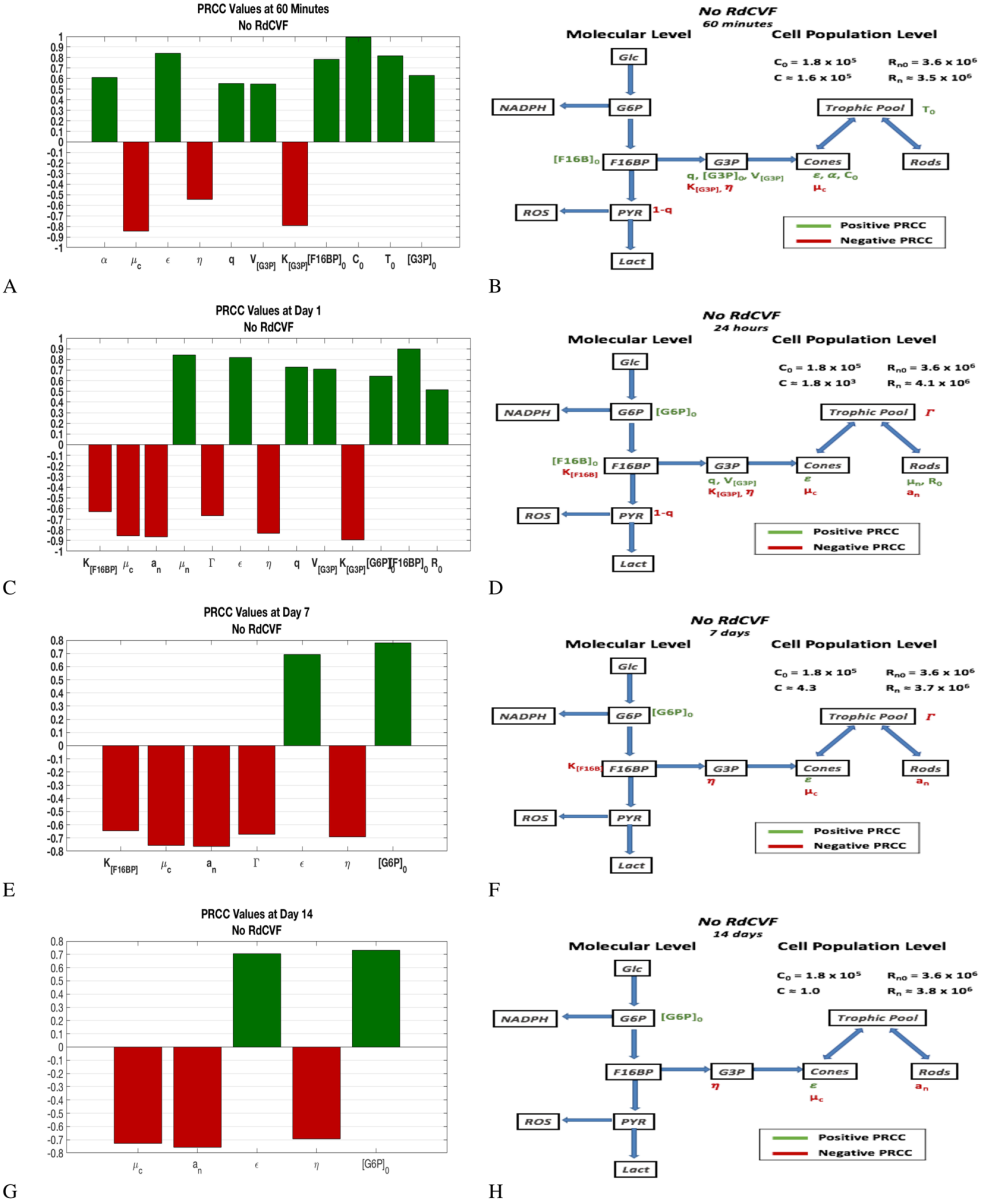
PRCC plots and corresponding effect on flow diagram in the absence of RdCVF. The two top panels, (A and B) are for 1 hour, the next two panels, (B and D), for 1 day, the next two panels, (E and F), for 7 days, and the two bottom panels, (G and H), are for 14 days. The left panels give the partial rank correlation coefficient (PRCC) plots for parameters with significant PRCC values while the right panels give the location of the significant PRCC quantity in the flow diagram. The value of *C* and *R*_*n*_ at the given time snapshot are given in each of the figures in the right panel and *C* is basically non-existent by day 7 in this scenario. The PRCC values are given in Table 18 (in Supplemental Information). See Sections 4.2 and 2.5 for details on PRCC analysis.

With no RdCVF available, two of the three processes (efficiency of OS disc production and competition by the rods for trophic) remain but the system is additionally influenced by upstream products. It is worth noting that *C* degenerates rapidly in this scenario and only a few cone OS remain by day 7 (from an initial level of 180,000). For early times (24 hours), it is the initial amount of G6P that matters together with the initial amount of F16BP and the *K*_*m*_ value of F16BP. At seven days, the initial amount of F16BP no longer matters and by 14 days, nothing within the production or use of F16BP has a large effect (with the Km value of F16BP no longer mattering); see Fig. 9 and Supplemental Information Table 18.

It is important to keep in mind that the analysis performed here looked at parameter ranges generated by considering ±10% changes from the parameter baseline values given in Table 2 with the exception of parameters that did not meet the monotonicity requirement; see Section 4 and Supplemental Information A.5. The only drastic changes in parameters were those that made the three cases: inefficient G3P use ($\epsilon$ is 3% of its normal value), drastically reduced RdCVF levels (*δ* is 0.1% of its normal value), and the absence of RdCVF ($\delta =0$).

## Discussion

The results presented here can be used to asses the biological significance of the parameters in relation to the cones to understand the driving mechanisms of cone viability in aerobic glycolysis within a cone photoreceptor. The developed mathematical model of the molecular and cellular level photoreceptor interactions consists of an 11-dimensional system of nonlinear ordinary differential equations. The 11-dimensional system is composed of two sub-systems; an 8-dimensional system that models the molecular level interactions within a single cone photoreceptor, and a 3-dimensional system that models the cellular level interactions between the rods, cones, and trophic pool.

Including only the committed (biochemically irreversible) aerobic glycolysis steps, the PPP, OXPHO, and phospholipid synthesis allowed us to focus on the most essential mechanisms of cone metabolism. Equally important was the incorporation of cellular level equations (describing the dynamics of cones, rods, trophic pool) that were modified and built from Camacho *et al*.^7,11,12^. Because the cell populations and molecular concentrations interact with and rely on each other, we are able to better understand how molecular level interactions affect cellular level populations. Mathematically, these interactions are illustrated via the equations where the outputs of each subsystem are included as inputs of the other system. Specifically, G3P is removed for phospholipid synthesis resulting in the renewal of cone OS, and thus the *C* equation depends on the concentration of G3P as the cone energy uptake, represented by equation (4), is a function of G3P. Similarly ROS has its own equation at the molecular level but since accumulation of ROS negatively affects the cones, it’s output [ROS](*t*) is an input to the cone equation, $\frac{dC}{dt}$. As another example, the rods synthesize RdCVF, which triggers the process of aerobic glycolysis in the cone photoreceptors by binding to a BSG1/GLUT1 complex, and accelerating the entry of glucose into the cell. To account for this, [*δR*_*n*_], the concentration of free unbound RdCVF, is included in the glucose (GLc) equation, $\frac{dg}{dt}$, in the molecular level subsystem.

Our model also includes two activation and inhibition functions, ${{\rm{\Omega }}}_{[{\rm{PYR}}]}$ and ${{\rm{\Phi }}}_{[{\rm{PYR}}]}$, at the molecular level. In the model, pyruvate was used as a proxy for accumulation of PEP due to oxidation of cysteine (358), a residue on pyruvate kinase. Thus, as ROS accumulates, PYR gets backed up. Once PYR reaches an equilibrium value, ${[{\rm{PEP}}]}_{eq}$, indicating that the concentration of ROS is too high, G6P re-routes glucose into the PPP and two molecules of NADPH are produced to help with the reduction of ROS. If PYR remains below ${[{\rm{PEP}}]}_{eq}$, F16BP is produced as glucose continues to travel down the lactate producing pathway. Thus, ${{\rm{\Omega }}}_{[{\rm{PYR}}]}$ and ${{\rm{\Phi }}}_{[{\rm{PYR}}]}$ are included in our $\frac{d[{\rm{G}}6{\rm{P}}]}{dt}$, $\frac{d[{\rm{F}}16{\rm{BP}}]}{dt}$, and $\frac{d[{\rm{NADPH}}]}{dt}$ equations. When $[{\rm{PYR}}] > {[{\rm{PYR}}]}_{eq}$, ${{\rm{\Phi }}}_{[{\rm{PYR}}]}$ is activated and ${{\rm{\Omega }}}_{[{\rm{PYR}}]}$ is inhibited promoting the creation of two NADPH molecules. When $[{\rm{PYR}}] < [{\rm{PYR}}{]}_{eq}$, ${{\rm{\Omega }}}_{[{\rm{PYR}}]}$ is activated and ${{\rm{\Phi }}}_{[{\rm{PYR}}]}$ is inhibited promoting the production of F16BP.

We considered the situation of a cone in which all processes were functioning properly and three different scenarios that showed various alterations from this. In the case of the cone in which all processes were functioning properly, we determined that three key sets of processes have the most effect on the cone OS production. There are two molecular processes: the contribution of F16BP to DHAP and GAP that ensure the reactions down the glycolysis pathway together with the availability of G3P and the efficiency to utilize G3P to make phospholipids. There is also a population-level process and it involves the cones and rods competition for resources. When we considered the effect of an inefficient use of glucose to make cone OS, the influence of the various parameters remained the same as in the case in which all processes were functioning properly.

The other two scenarios involved RdCVF. In the extreme case of no RdCVF, *C* dies off as the cone OS production cannot be maintained by glucose uptake in the absence of RdCVF. When there is a limited amount of RdCVF, the efficiency of making phospholipids and competition by the rods for resources remain important processes in cone OS production. Additionally, we found that the intracellular levels of glucose significantly affect the system. Taken together, in situations in which there are low levels of rods as in the case of RP, it is crucial to ensure there are sufficient levels of RdCVF and trophic factors available in order to maintain cone OS renewal.

The Michaelis-Menten and allosteric kinetics are well-documented ways to observe individual chemical reactions as described in the manuscript. Thus, we believe the predictions of strength and importance of various pathways suggested by our results can give important guidance to experimentalists trying to identify such key pathways. The goal of this paper was to verify the reliance of rods on cones via RdCVF and understand which processes (represented by the parameters) contribute to the vitality of the cones using uncertainty and sensitivity analysis.

To the authors’ knowledge, this is the first time that an analysis of aerobic glycolysis in a cone photoreceptor has been conducted. We used the outcome of our sensitivity analysis to help identify which parameters have the greatest affect on the cones. These results can lead to important insight into the design of new experiments and at what time point a particular parameter’s affect on the cones should be considered.

## Methods

### Mathematical Model

Following the descriptions in Sections 2.1, 2.2, and 2.3, we have the following equations that govern the dynamics of the system at the molecular and cellular levels.

Molecular level:

5 $$\frac{d[{\rm{g}}]}{dt}=\lambda ([G]-[{\rm{g}}])\,(\frac{{V}_{{{\rm{\max }}}_{[{\rm{g}}]}}{(\delta {R}_{n})}^{2}}{{K}_{{m}_{[{\rm{g}}]}}^{2}+{(\delta {R}_{n})}^{2}}+p)-\frac{{V}_{{{\rm{\max }}}_{[{\rm{G}}6{\rm{P}}]}}{[{\rm{g}}]}^{2}}{{K}_{{m}_{[{\rm{G}}6{\rm{P}}]}}^{2}+{[{\rm{g}}]}^{2}}$$

6 $$\tfrac{d[{\rm{G}}6{\rm{P}}]}{dt}=\tfrac{{V}_{{{\rm{\max }}}_{[\mathrm{G6P}]}}{[{\rm{g}}]}^{2}}{{K}_{{m}_{[{\rm{G}}6{\rm{P}}]}}^{2}+{[{\rm{g}}]}^{2}}-\tfrac{{V}_{{{\rm{\max }}}_{[{\rm{F}}16{\rm{BP}}]}}{[{\rm{G6P}}]}^{2}}{{K}_{{m}_{[{\rm{F}}16{\rm{BP}}]}}^{2}+{[{\rm{G}}6{\rm{P}}]}^{2}}{{\rm{\Omega }}}_{[{\rm{PYR}}]}-\tfrac{2{V}_{{{\rm{\max }}}_{[{\rm{NADPH}}]}}{[{\rm{G6P}}]}^{2}}{{K}_{{m}_{[{\rm{NADPH}}]}}^{2}+{[{\rm{G6P}}]}^{2}}{{\rm{\Phi }}}_{[{\rm{PYR}}]}$$

7 $$\tfrac{d[{\rm{F}}16{\rm{BP}}]}{dt}=\tfrac{{V}_{{{\rm{\max }}}_{[{\rm{F}}16{\rm{BP}}]}}{[{\rm{G}}6{\rm{P}}]}^{2}}{{K}_{{m}_{[{\rm{F}}16{\rm{BP}}]}}^{2}+{[{\rm{G}}6{\rm{P}}]}^{2}}{{\rm{\Omega }}}_{[{\rm{PYR}}]}-\tfrac{(1-q)\,{V}_{{{\rm{\max }}}_{[{\rm{PYR}}]}}{[{\rm{F}}16{\rm{BP}}]}^{2}}{{K}_{{m}_{[{\rm{PYR}}]}}^{2}+{[{\rm{F}}16{\rm{BP}}]}^{2}}-\tfrac{q{V}_{{{\rm{\max }}}_{[{\rm{G}}3{\rm{P}}]}}{[{\rm{F}}16{\rm{BP}}]}^{2}}{{K}_{{m}_{[{\rm{G}}3{\rm{P}}]}}^{2}+{[{\rm{F}}16{\rm{BP}}]}^{2}}$$

8 $$\frac{d[{\rm{G}}3{\rm{P}}]}{dt}=\frac{q{V}_{{{\rm{\max }}}_{[{\rm{G}}3{\rm{P}}]}}{[{\rm{F}}16{\rm{BP}}]}^{2}}{{K}_{{m}_{[{\rm{G}}3{\rm{P}}]}}^{2}+{[{\rm{F}}16{\rm{BP}}]}^{2}}-\eta \alpha C[{\rm{G}}3{\rm{P}}]$$

9 $$\frac{d[{\rm{NADPH}}]}{dt}=\frac{2{V}_{{{\rm{\max }}}_{[{\rm{NADPH}}]}}{[{\rm{G}}6{\rm{P}}]}^{2}}{{K}_{{m}_{[{\rm{NADPH}}]}}^{2}+{[{\rm{G}}6{\rm{P}}]}^{2}}{{\rm{\Phi }}}_{[{\rm{PYR}}]}-\tau [{\rm{NADPH}}]$$

10 $$\frac{d[{\rm{PYR}}]}{dt}=\frac{(1-q)\,{V}_{{{\rm{\max }}}_{[{\rm{PYR}}]}}{[{\rm{F}}16{\rm{BP}}]}^{2}}{{K}_{{m}_{[{\rm{PYR}}]}}^{2}+{[{\rm{F}}16{\rm{BP}}]}^{2}}-\frac{(1-\rho )\,{V}_{{{\rm{\max }}}_{[{\rm{LACT}}]}}[{\rm{PYR}}]}{{K}_{{m}_{[{\rm{LACT}}]}}+[{\rm{PYR}}]}$$

11 $$\frac{d[{\rm{LACT}}]}{dt}=\frac{(1-\rho )\,{V}_{{{\rm{\max }}}_{[{\rm{LACT}}]}}[{\rm{PYR}}]}{{K}_{{m}_{[{\rm{LACT}}]}}+[{\rm{PYR}}]}-\psi [{\rm{LACT}}]$$

12 $$\frac{d[{\rm{ROS}}]}{dt}=\frac{\rho [{\rm{PYR}}]}{1+\omega [{\rm{NADPH}}]}+r-{\delta }_{L}[{\rm{ROS}}]$$

Cellular (population) level:

13 $$\frac{dC}{dt}=CT\epsilon \alpha [{\rm{G}}3{\rm{P}}]-C{\mu }_{c}-C{\mu }_{[{\rm{ROS}}]}[{\rm{ROS}}]$$

14 $$\frac{d{R}_{n}}{dt}={R}_{n}T{a}_{n}-{R}_{n}{\mu }_{n}$$

15 $$\frac{dT}{dt}=T({\rm{\Gamma }}-kT-{\beta }_{n}{R}_{n}-\gamma C)$$

with variables and parameters previously defined and parameter values given in Table 2 and Supplemental Information A.1 and A.2.

### Uncertainty and Sensitivity Analysis

Uncertainty and sensitivity analyses are necessary to explore how uncertainty in the input parameter values affects the model outputs. In any mathematical model of a complex system one is bound to have uncertainties in the input parameters and these will be reflected in the output of the model. This uncertainty can be attributed to errors, noise, or variability in the experimental data from which parameter values are inferred or due to the fact that exact parameter values are unknown or cannot be inferred from the data. Sensitivity analysis (SA) measures the change in the output brought about by a unit change in a particular input parameter or initial condition. It allows us to determine the contributions of the uncertain inputs to the uncertainty in the analysis results^32^. Both uncertainty and sensitivity analysis go hand in hand because the analysis and results of the model outputs are functions of the input parameters and their respective uncertainties^35,36^. In our uncertainty analysis (UA), inputs are generated by the Latin Hypercube Sampling (LHS) procedure.

To implement LHS, all parameters were set to a baseline value (given in Table 2) and then the parameter sample space was generated over a [−10%, +10%] variation from the baseline. For parameter values in which a change did not yield a monotonic change in the cone values, we restricted their respective ranges as monotonicity is one of the criteria of the methodology; see Fig. 11 in the Supplemental Material. This occurred in one to three parameters in the SA for the three different cases we investigated and is described in Supplemental Information A.5. Furthermore, there were a few other parameters held constant in the SA code because the corresponding percent change in cones was insignificant; see Supplemental Information A.5. For LHS implementation every parameter space was partitioned into non-overlapping intervals of equal probability and assigned a probability density function (pdf) according to a uniform probability distribution. Exactly one value was sampled/selected at random from each interval with respect to the pdf over the parameter space.

PRCC analysis is used when there exists a nonlinear, monotonic relationship between inputs and outputs^35^. Rather than measuring the correlation between the variable values, the PRCC value measures the correlation of the ranked ordering of the variable values^37^. PRCC values are between −1 and 1. The magnitude of the PRCC indicates the sensitivity of the output to the parameter value uncertainty, and the sign of the PRCC value indicates that either a positive or negative correlation exists. Using the standard threshold values, |PRCC| > 0.5 and p-value < 0.05, where the p-value determines the significance of the PRCC value, we may say that the output is sensitive to the parameter^35^. This method described provides a global UA and SA to understand the effect of the inputs into the system^35^.

## Supplementary information

Supplementary Info

## References

[1] Wong-Riley M (2010). Energy metabolism of the visual system. Eye and Brain.

[2] Young R (1967). The renewal of photoreceptor cell outer segments. J. Cell Biol..

[3] O’Day W, Young R (1978). Rhythmic daily shedding of outer-segment membranes by visual cells in the goldfish. J. Cell Biol..

[4] Zhang Y, Chioreso C, Schweizer ML, Abramoff MD (2017). Effects of aflibercept for neovascular age-related macular degeneration: a systematic review and meta-analysis of observational comparative studies. Invest. Ophth. Vis. Sci..

[5] Camacho ET, Melara LA, Villalobos MC, Wirkus S (2014). Optimal control in the treatment of retinitis pigmentosa. B. Math. Biol..

[6] Léveillard T (2004). Identification and characterization of rod-derived cone viability factor. Nat. Genet..

[7] Camacho ET, Punzo C, Wirkus SA (2016). Quantifying the metabolic contribution to photoreceptor death in retinitis pigmentosa via a mathematical model. J. Theor. Biol..

[8] Léveillard T, Sahel J-A (2010). Rod-derived cone viability factor for treating blinding diseases: from clinic to redox signaling. Sci. Transl. Med..

[9] Léveillard T, Sahel J-A (2017). Metabolic and redox signaling in the retina. Cell. Mol. Life Sci..

[10] Aït-Ali N (2015). Rod-derived cone viability factor promotes cone survival by stimulating aerobic glycolysis. Cell.

[11] Camacho ET (2010). A mathematical model for photoreceptor interactions. J. Theor. Biol..

[12] Camacho ET, Wirkus S (2013). Tracing the progression of retinitis pigmentosa via photoreceptor interactions. J. Theor. Biol..

[13] Camacho ET, Léveillard T, Sahel J-A, Wirkus S (2016). Mathematical model of the role of RdCVF in the coexistence of rods and cones in a healthy eye. B. Math. Biol..

[14] Nohl H, Gille L, Staniek K (2005). Intracellular generation of reactive oxygen species by mitochondria. Biochem. Pharmacol..

[15] Mei X (2016). The thioredoxin encoded by the rod-derived cone viability factor gene protects cone photoreceptors against oxidative stress. Antioxid. Redox Sign..

[16] Kim D-I (2015). Hyperglycemia-induced GLP-1R downregulation causes RPE cell apoptosis. Int. J. Biochem. Cell B..

[17] Hebert DN, Carruthers A (1992). Glucose transporter oligomeric structure determines transporter function. Reversible redox-dependent interconversions of tetrameric and dimeric GLUT1. J. Biol. Chem..

[18] Moretti AIS, Laurindo FRM (2017). Protein disulfide isomerases: Redox connections in and out of the endoplasmic reticulum. Arch. Biochem. Biophys..

[19] Berg, J. M., L., T. J. & Stryer, L. *Biochemistry* (Palgrave MacMillan; 7th revised international ed edition, 2011).

[20] Grüning N-M, Du D, Keller MA, Luisi BF, Ralser M (2014). Inhibition of triosephosphate isomerase by phosphoenolpyruvate in the feedback-regulation of glycolysis. Open Biol..

[21] Chinchore Y, Begaj T, Wu D, Drokhlyansky E, Cepko CL (2017). Glycolytic reliance promotes anabolism in photoreceptors. Elife.

[22] Kanow MA (2017). Biochemical adaptations of the retina and retinal pigment epithelium support a metabolic ecosystem in the vertebrate eye. Elife.

[23] Hurley JB, Lindsay KJ, Du J (2015). Glucose, lactate, and shuttling of metabolites in vertebrate retinas. J. Neurosci. Res..

[24] Stone J (1999). Mechanisms of photoreceptor death and survival in mammalian retina. Prog. Retin. Eye Res..

[25] Fridlich R (2009). The thioredoxin-like protein rod-derived cone viability factor (RdCVFL) interacts with TAU and inhibits its phosphorylation in the retina. Mol. Cell. Proteomics.

[26] Zhang, F., Liu, Z., Kurokawa, K. & Miller, D. T. *Tracking dynamics of photoreceptor disc shedding with adaptive optics*-*optical coherence tomography* in *Ophthalmic Technologies XXVII* **10045**, 1004517 (2017).

[27] Wolloscheck, T., Kunst, S., Kelleher, D. K. & Spessert, R. Transcriptional regulation of nucleoredoxinlike genes takes place on a daily basis in the retina and pineal gland of rats. *Visual Neurosci*. **32** (2015). 10.1017/S0952523814000352 26239254

[28] Delyfer, M.-N. *et al*. Transcriptomic Analysis of Human Retinal Surgical Specimens Using jouRNAl. *Jove*-*J*. *Vis*. *Exp*. **78** (2013). 10.3791/50375 PMC3846914 23979175

[29] Law A-L (2015). Cleavage of mer tyrosine kinase (MerTK) from the cell surface contributes to the regulation of retinal phagocytosis. J. Biol. Chem..

[30] Clérin E (2011). **e**-conome: an automated tissue counting platform of cone photoreceptors for rodent models of retinitis pigmentosa. BMC Ophthalmol..

[31] Helton JC, Davis F (2002). Illustration of sampling-based methods for uncertainty and sensitivity analysis. Risk Anal..

[32] Helton JC, Johnson JD, Sallaberry CJ, Storlie CB (2006). Survey of sampling-based methods for uncertainty and sensitivity analysis. Reliab. Eng. Syst. Safe..

[33] Byrne LC (2015). Viral-mediated RdCVF and RdCVFL expression protects cone and rod photoreceptors in retinal degeneration. J. Clin. Invest..

[34] Hanein, S. *et al*. In, 9–14 (Springer, 2006).

[35] Marino S, Hogue IB, Ray CJ, Kirschner DE (2008). A methodology for performing global uncertainty and sensitivity analysis in systems biology. J. Theor. Biol..

[36] Blower, S. M. & Dowlatabadi, H. Sensitivity and uncertainty analysis of complex models of disease transmission: an HIV model, as an example. *Int*. *Stat*. *Rev*. 229–243 (1994).

[37] Conover WJ, Iman RL (1981). Rank transformations as a bridge between parametric and nonparametric statistics. Am. Stat..

[38] Carruthers, A. GLUT1 Structure, Function and Trafficking-Regulation by Cellular Redox and Metabolic Status. *Metabolic and Redox Signalling in the Retina and Central Nervous System*, http://www.college-de-france.fr/site/en-jose-alain-sahel/studyday-2016-03-16-14h45.htm (Accessed: 10-27-2016).

[39] Marín-Hernández A (2014). Modeling cancer glycolysis under hypoglycemia, and the role played by the differential expression of glycolytic isoforms. FEBS J..

[40] Moreno-Sánchez R (2012). Phosphofructokinase type 1 kinetics, isoform expression, and gene polymorphisms in cancer cells. J. Cell. Biochem..

[41] Rufino-Palomares EE (2016). NADPH production, a growth marker, is stimulated by maslinic acid in gilthead sea bream by increased NADP-IDH and ME expression. Comp. Biochem. Phys. C..

[42] Kashiwaya Y (1994). Control of glucose utilization in working perfused rat heart. J. Biol. Chem..

[43] Zhang L (2014). A polymer-based ratiometric intracellular glucose sensor. Chem. Commun..

