# Supplementary material for: A Mathematical Analysis of Aerobic Glycolysis Triggered by Glucose Uptake in Cones
Source: Sci Rep. 2019 Mar 11;9:4162. doi: 10.1038/s41598-019-39901-z (PMC6411757; doi:10.1038/s41598-019-39901-z)
Supplement: Supplementary file 1 — Supplementary Info [file 41598_2019_39901_MOESM1_ESM.pdf]

Supplemental Information for A Mathematical Analysis of Aerobic Glycolysis Triggered by Glucose Uptake in Cones

Erika T. Camacho[1,*], Danielle Brager[2], Ghizlane Elachouri[3], Tatyana Korneyeva[1], Géraldine Millet-Puel[3], José-Alain Sahel[3], Thierry Léveillard[3]

A Appendix

A.1 Conversion of $V\text{max}_i$ Values

Several $V\text{max}_i$ values in Table 2 in the main text were obtained from the literature, and the units needed to be converted to $\text{mM}\cdot\text{min}^{-1}$. The units were converted using the conversion factor of volume per density listed in the source literature.

For example, consider $V\text{max}_{[\text{G6P}]} = 41 \pm 8\ \text{nmol}\cdot\text{min}^{-1}\cdot\text{mg}^{-1}$. Using the conversion factor, $4.5\ \text{mg}\cdot\text{ml}^{-1}$, we obtain

$$V\text{max}_{[\text{G6P}]} = \left(41 \pm 8\ \frac{\text{nmol}}{\text{min}\cdot\text{mg}}\right)\left(\frac{10^{-6}\ \text{mmol}}{1\ \text{nmol}}\right)\left(4.5\ \frac{\text{mg}}{\text{ml}}\right)\left(\frac{1000\ \text{ml}}{1\ \text{l}}\right)$$
$$= 0.1845 \pm 0.036\ \text{mM}\cdot\text{min}^{-1}.$$

The table below contains the source parameter values as well as the conversion factors used to convert the units to $\text{mM}\cdot\text{min}^{-1}$.

Table 3: Table of converted $V\text{max}_\text{i}$ values.

Parameter	Source Value	Conversion Factor	Value in Table 2	Source
$V\text{max}_{[\text{G6P}]}$	$41 \pm 8\ \text{nmol}\cdot\text{min}^{-1}\cdot\text{mg}^{-1}$	$4.5\ \text{mg}\cdot\text{mL}^{-1}$	$0.1845 \pm 0.036\ \text{mM}\cdot\text{min}^{-1}$	[39]
$V\text{max}_{[\text{F16B}]}$	$21 \pm 7\ \text{nmol}\cdot\text{min}^{-1}\cdot\text{mg}^{-1}$	$65\ \text{mg}\cdot\text{mL}^{-1}$	$1.365 \pm 0.455\ \text{mM}\cdot\text{min}^{-1}$	[40]
$V\text{max}_{[\text{PYR}]}$	$87 \pm 41\ \text{nmol}\cdot\text{min}^{-1}\cdot\text{mg}^{-1}$	$4.5\ \text{mg}\cdot\text{mL}^{-1}$	$0.3915 \pm 0.1845\ \text{mM}\cdot\text{min}^{-1}$	[39]
$V\text{max}_{[\text{NADPH}]}$	$2.28 \pm 0.13\ \text{mU}$	$10^{-1}\ \text{g}\cdot\text{mL}^{-1}$	$0.228 \pm 0.013\ \text{mM}\cdot\text{min}^{-1}$	[41]

This is a table of $V\text{max}$ values obtained from the sources listed in the source column. The $V\text{max}$ values listed had units that needed to be converted to $\text{mM}\cdot\text{min}^{-1}$ using a conversion factor. Each $V\text{max}$ in this table had a corresponding conversion factor found in the same source file. The sources can be found in the main text. Table 2 is the table of parameters, and can be found in the main text.

A.2 Description of Our Experimental Parameter Values

The parameter values with an asterisk (*) in Table 2 (in the main text) are our experimental values. In the caption of the table, we provided a short description of how those values were obtained. In the paragraph below, we provide more details.

Cone-enriched cultures made of the retina of chicken embryo was analyzed after four days in vitro [10]. Cells from a dish of 60 mm of diameter were washed with phosphate buffer saline (PBS). The PBS was removed and the cells were frozen on liquid nitrogen to freeze enzymatic reactions. To each cell 5 ml of acetonitrile / methanol /water (4: 4: 2 vol/vol) at -20°C were added and 120 μl of internal standard, a ^{13}C *E. Coli* metabolome reference for isotopic dilution before mass spectrometry. The cells were scraped, vortexed, and frozen at -80°C prior to the metabolomic analysis. Metabolites were separated by ionic chromatography (Dionex) and were detected using mass spectrometry (QTrap 4000, Applied Biosystems) using electrospray ionization (negative mode) and multiple reaction monitoring. Quantified values were obtained by referring to the E.Coli ^{13}C metabolome as internal standard.

A.3 RdCVF and RdCVFL Expression Data

We referred to rod-derived cone viability factor (RdCVF) and rod-derived cone viability factor long (RdCVFL) expression data from our experimental work in Figure 5(located in Section 2.4 in the main text). We have included an explanation of the collection of that data below, and the corresponding figures (see Figure 10 below).

Wild-type mice (BALB/cJ) were housed under the 12-hour light/12-hour dark cycle, 23-25°C at room temperature, and offered ad libitum access to food and water. The project was approved by the French Ethics Committee (No. A-75-1863; OGM No. 5080 CA-II), and all experiments were performed in accordance with the European Community Council Directives of September 22, 2010 (2010/63/UE). Groups of 2 males and 2 females have been sacrificed every 4 hours (0, 4, 8, 12, 16 and 20 hours). Neural retina were dissected and the ribonucleic acid (RNA) purified using cesium chloride (CsCl) ultracentrifugation [28]. 500 ng of RNA were used for complementary deoxyribonucleic acid (cDNA) reverse transcription (Superscript III enzyme) with random hexamer. Quantitative reverse transcription polymerase chain reaction (RT-PCR) was performed with the following primers: Glyceraldehyde 3-phosphate dehydrogenase (*Gapdh*) (5'-TGGTGAAGGTCGGTGTGAACGGAT-3', 5'-TCCATGGTGGTGAAGACACCAGTA-3'), rod-derived cone viability factor (RdCVF) messenger RNA (mRNA) (5'-CTACAGAGGAGCAACAGGAC-3', 5'-TGCACAAGTAGTACCAGGAC-3') and rod-derived cone viability factor long (RdCVFL) mRNA (5'-GCAACAGGACCTCTTCCTCA-3', 5'-CCAGACGCTGGATCTCCTC-3'). Expression data was normalized to *Gapdh*. The data are plotted as percentage expression to the maximal value; see Figure 10.

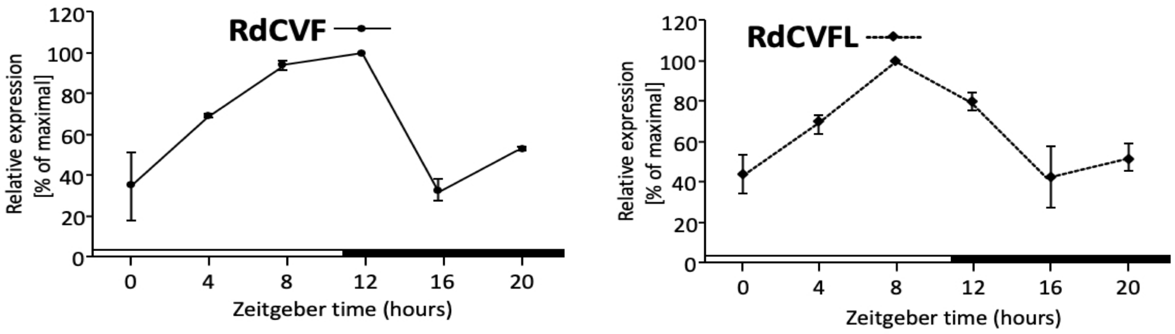

A B

Figure 10: RdCVF and RdCVFL expression data. RdCVF and RdCVFL expression data was collected as a part of our experimental work. Quantitative reverse transcription polymerase chain reaction (RT-PCR) was performed and the data are plotted as percentage of expression to the maximal value.

A.4 Analysis of Four Cases

Simulations for sensitivity analysis (SA), run in MATLAB using ODE15s with RelTol $= 2.3 \times 10^{-14}$ and 500 runs, were made for the Latin Hypercube Sampling (LHS) procedure. All parameters are as in Table 2 in the main text unless otherwise stated. We considered four cases:

1. All processes functioning properly
2. Inefficient use of glucose for cone outer segment (OS) renewal ($\epsilon = 9.99 \times 10^{-4}$, i.e., 3% of its normal value)
3. Insufficient rod-derived cone viability factor (RdCVF) ($\delta = 65 \times 10^{-9}$, i.e., 0.1% of its normal value)
4. No RdCVF ($\delta = 0$)

We define the proportion of full length of an OS to be its current length divided by the maximum OS length. The proportion of full length of each OS fluctuates throughout the day due to periodic shedding and continuous renewal such that at any time an OS proportion of full length can take any value between 0 and 1. In a healthy retina this value would be far away from zero. Thus, we let C represent the sum of the proportion of full length of each cone OS in the retina. Similarly, we let R_n represent the sum of the proportion of full length of each rod OS in the retina.

Table 4: Simulation comparisons for each case at 60 minutes.

Case	C Value	R_n Value	Ratio of R_n to C
Healthy	1.82×10^5	3.52×10^6	19.36
Small ϵ	1.47×10^5	3.52×10^6	23.91
Small δ	1.64×10^5	3.52×10^6	21.46
No δ	1.64×10^5	3.52×10^6	21.46

Table 5: Simulation comparisons for each case at 24 hours.

Case	C Value	R_n Value	Ratio of R_n to C
Healthy	1.96×10^5	4.09×10^6	20.92
Small ϵ	7.03×10^3	4.11×10^6	584.37
Small δ	5.14×10^3	4.11×10^6	779.82
No δ	1.79×10^3	4.11×10^6	2296.37

Table 6: Simulation comparisons for each case at day 7.

Case	C Value	R_n Value	Ratio of R_n to C
Healthy	1.91×10^5	3.74×10^6	19.58
Small ϵ	5.70×10^3	3.71×10^6	650.60
Small δ	3.51×10^3	3.71×10^6	1059.03
No δ	4.27	3.71×10^6	870274.87

Table 7: Simulation comparisons for each case at day 14.

Case	C Value	R_n Value	Ratio of R_n to C
Healthy	1.93×10^5	3.89×10^6	20.14
Small ϵ	5.74×10^3	3.82×10^6	665.66
Small δ	3.57×10^3	3.82×10^6	1070.81
No δ	1.04	3.82×10^6	$3,675,538.61$

A.5 Parameter Space Restriction in Uncertainty Analysis and Sensitivity Analysis

Case of All Processes Functioning Properly

Table 8: Parameter space restrictions at $t = 7, 14$ days when **all processes functioning properly**. No parameter restriction is needed for $t = 60$ minutes or $t = 1$ day in the latin hypercube sampling/partial rank correlation coefficient (LHS/PRCC) sensitivity analysis procedure to satisfy monotonicity.

	$t = 7$ Min. Value	$t = 7$ Max. Value	$t = 14$ Min. Value	$t = 14$ Max. Value	Baseline Value
Γ	2.05×10^{-3}	2.3×10^{-3}	2.18×10^{-3}	2.27×10^{-3}	2.22×10^{-3}
μ_n	5.6×10^{-3}	6.3×10^{-3}	6.2×10^{-3}	6.45×10^{-3}	6.25×10^{-3}
T_0	no restriction	no restriction	8.25×10^4	9.2×10^4	8.3×10^4

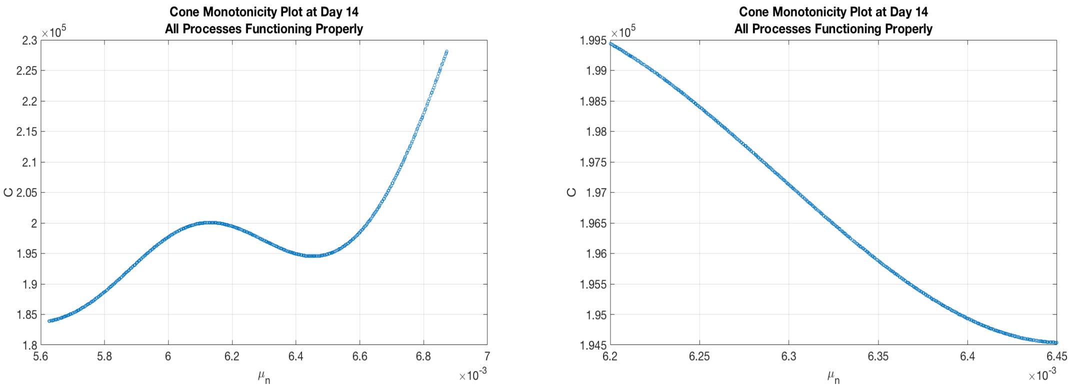

A B

Figure 11: Monotonicity plots: Graphical illustration of parameter space restrictions. Panel A for the parameter space of [μ_n -%10, μ_n +%10] and Panel B for the restricted parameter space of μ_n for $t = 14$ days when all processes functioning properly.

Table 9: Parameters **held constant** at their baseline value $t = 60$ minutes or $t = 1, 7, 14$ days for **all processes functioning properly** in the LHS/PRCC sensitivity analysis procedure to satisfy monotonicity. These parameters are held constant in the sensitivity analysis code because the corresponding cone, C, percent change was extremely small.

Parameter	Baseline Value	C % Change $t = 60$	C % Change $t = 1$	C % Change $t = 7$	C % Change $t = 14$
$[\text{LACT}]_0$	1×10^{-4}	2.63×10^{-11}	7.01×10^{-10}	1.06×10^{-9}	1.01×10^{-9}
$[\text{NADPH}]_0$	1.2×10^{-4}	1.66×10^{-10}	6.25×10^{-10}	4.64×10^{-10}	4.79×10^{-10}
Ψ	8	2.63×10^{-11}	7.01×10^{-10}	1.07×10^{-9}	8.10×10^{-10}

Case of Inefficient Use of Glucose by Cones

Table 10: Parameter **space restrictions** at $t = 7, 14$ days for **inefficient use of glucose for cone OS renewal.** No parameter restriction is needed for $t = 60$ minutes or $t = 1$ day in the LHS/PRCC sensitivity analysis procedure to satisfy monotonicity.

	t=7 Min. Value	t=7 Max. Value	t=14 Min. Value	t=14 Max. Value	Baseline Value
Γ	2×10^{-3}	2.27×10^{-3}	2.13×10^{-3}	2.27×10^{-3}	2.22×10^{-3}
μ_n	5.6×10^{-3}	6.3×10^{-3}	6.1×10^{-3}	6.4×10^{-3}	6.25×10^{-3}
T_0	no restriction	no restriction	8.1×10^4	9.2×10^4	8.3×10^4

Table 11: Parameter **held constant** at $t = 60$ minutes and $t = 1, 7, 14$ days for **inefficient use of glucose for cone OS renewal** ($\epsilon = 1 \times 10^{-3}$)**.** These parameters are held constant in the sensitivity analysis code because the corresponding cone, C, percent change was extremely small.

Parameter	Baseline Value	C % Change $t = 60$	C % Change $t = 1$	C % Change $t = 7$	C % Change $t = 14$
$[\text{LACT}]_0$	1×10^{-4}	2.66×10^{-11}	1.29×10^{-9}	2.96×10^{-9}	2.49×10^{-9}
$[\text{NADPH}]_0$	1.2×10^{-4}	9.51×10^{-11}	1.07×10^{-9}	2.60×10^{-9}	2.19×10^{-9}
Ψ	8	5.69×10^{-11}	1.30×10^{-9}	2.83×10^{-9}	2.42×10^{-9}

Case of Insufficient RdCVF

Table 12: Parameter **space restrictions** at $t = 60$ minutes and $t = 7, 14$ days for **insufficient RdCVF** ($\delta = 65 \times 10^{-9}$)**.** No parameter restriction is needed for $t = 1$ day in the LHS/PRCC sensitivity analysis procedure to satisfy monotonicity.

	$t = 60$ Min. Value	$t = 60$ Max Value	$t = 7$ Min. Value	$t = 7$ Max Value	$t = 14$ Min. Value	$t = 14$ Max Value	Baseline Value
Γ	no restriction	no restriction	2×10^{-3}	2.24×10^{-3}	2.05×10^{-3}	2.23×10^{-3}	2.22×10^{-3}
μ_n	no restriction	no restriction	5.6×10^{-3}	6.4×10^{-3}	5.6×10^{-3}	6.3×10^{-3}	6.25×10^{-3}
R_{n_0}	3.8×10^6	4.3×10^6	no restriction	no restriction	no restriction	no restriction	3.6×10^6

Table 13: Parameters **held constant** for $t = 14$ days in the case when there is **insufficient RdCVF** ($\delta = 65 \times 10^{-9}$) for the sensitivity analysis because the corresponding cone percent change was almost negligible. There are no parameters that need to be held constant in the LHS/PRCC sensitivity analysis procedure to satisfy monotonicity for $t = 60$ minutes or $t = 1, 7$ days in the case when there is insufficient RdCVF.

Parameter	Baseline Value	Cone Percent Change
$[\text{NADPH}]_0$	1.2×10^{-4}	1.85×10^{-11}
$[\text{PYR}]_0$	1×10^{-4}	2.13×10^{-11}
$[\text{ROS}]_0$	8	2.07×10^{-11}

Case of No RdCVF

Table 14: Parameter **space restrictions** at $t = 7, 14$ days for **no RdCVF**. No parameter restriction is needed for $t = 60$ minutes or $t = 1$ day in the LHS/PRCC sensitivity analysis procedure to satisfy monotonicity.

	$t = 7$ Min. Value	$t = 7$ Max. Value	$t = 14$ Min. Value	$t = 14$ Max. Value	Baseline Value
Γ	2×10^{-3}	2.26×10^{-3}	2.12×10^{-3}	2.26×10^{-3}	2.22×10^{-3}
μ_n	6.15×10^{-3}	6.45×10^{-3}	6.1×10^{-3}	6.42×10^{-3}	6.25×10^{-3}
T_0	no restriction	no restriction	8.1×10^4	9.2×10^4	8.4×10^4

No parameters needed to be held constant in the LHS/PRCC sensitivity analysis procedure to satisfy monotonicity for $t = 60$ minutes or day 1, 7, and 14 for the case in which there is *no RdCVF*.

A.6 PRCC Values of Specific Cases

Case of All Processes functioning Properly

Table 15: PRCC Data for parameters with $|\text{PRCC}| > 0.5$ and p-value < 0.05. **All processes functioning properly**. These PRCC values correspond to Figure 7 in the main text.

Parameter	PRCC 60 Min.	PRCC Day 1	PRCC Day 7	PRCC Day 14
$V\text{max}_{[\text{PYR}]}$	-0.598	-0.648	-0.566	-0.575
$K_{m_{[\text{PYR}]}}$	0.832	0.853	0.820	0.828
$V\text{max}_{[\text{LACT}]}$	0.615	0.586	0.551	0.621
$K_{m_{[\text{LACT}]}}$	-0.577	-0.653	-0.544	-0.590
μ_c	-0.739	-0.773	-0.696	-0.698
a_n		-0.815	-0.810	-0.612
μ_n		0.791	0.598	
Γ		-0.548		
ϵ	0.729	0.741	0.698	0.725
η	-0.725	-0.780	-0.710	-0.682
q	0.726	0.770	0.700	0.719
$V\text{max}_{[\text{G3P}]}$	0.702	0.702	0.648	0.638
$K_{m_{[\text{G3P}]}}$	-0.830	-0.859	-0.818	-0.813

Case of Inefficient Use of Glucose by Cones

Table 16: PRCC Data for parameters with $|\text{PRCC}| > 0.5$ and p-value < 0.05. **Inefficient use of glucose for cone OS renewal**. These PRCC values correspond to Figure 12 below.

Parameter	PRCC 60 Min	PRCC Day 1	PRCC Day 7	PRCC Day 14
$V\text{max}_{[\text{PYR}]}$	-0.659	-0.660	-0.640	-0.627
$K_{m_{[\text{PYR}]}}$	0.849	0.853	0.841	0.828
$V\text{max}_{[\text{LACT}]}$	0.648	0.604	0.603	0.568
$K_{m_{[\text{LACT}]}}$	-0.605	-0.595	-0.613	-0.570
μ_c	-0.774	-0.749	-0.720	-0.732
a_n		-0.788	-0.846	-0.765
μ_n		0.763	0.656	
Γ		-0.506		
ϵ	0.761	0.751	0.725	0.720
η	-0.778	-0.725	-0.717	-0.718
q	0.785	0.747	0.759	0.760
$V\text{max}_{[\text{G3P}]}$	0.726	0.679	0.695	0.681
$K_{m_{[\text{G3P}]}}$	-0.859	-0.843	-0.847	-0.833

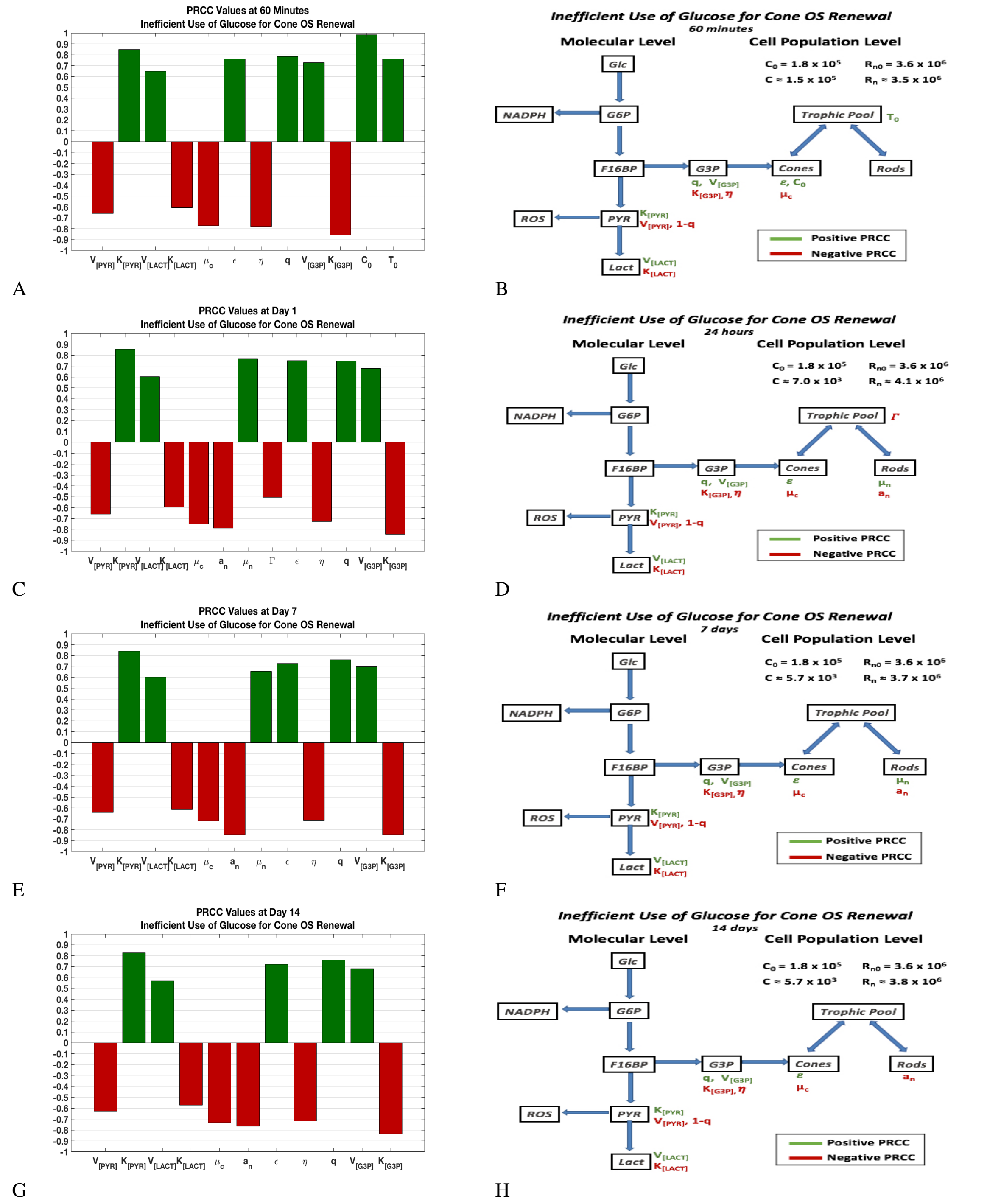

Figure 12: PRCC plots and corresponding effect on flow diagram for inefficient use of glucose. The two top panels, A and B are for 1 hour, the next two panels, B and D, for 1 day, the next two panels, E and F, for 7 days, and the two bottom panels, G and H, are for 14 days. The left panels give the PRCC plots for parameters with significant PRCC values while the right panels give the location of the significant PRCC quantity in the flow diagram. The value of C and R_n at the given time snapshot are given in each of the figures in the right panel. The value of C is significantly reduced but levels off to a reduced value. The PRCC values are given in Table 16. The parameters that are significant in this case are also significant when all processes are functioning properly; see Figure 7 in the main text. See Sections Sections 4.2 and 2.5 in the main text for details on PRCC analysis.

Case of Insufficient RdCVF

Table 17: PRCC Data for parameters with |PRCC| > 0.5 and p-value < 0.05. **Insufficient RdCVF**. These PRCC values correspond to Figure 8 in the main text.

Parameter	PRCC 60 Min	PRCC Day 1	PRCC Day 7	PRCC Day 14
$[G]$		0.630		
$V\text{max}_{[\text{g}]}$		0.637		
δ		0.839	0.690	0.706
$K_{m_{[\text{g}]}}$		−0.845	−0.681	−0.671
$V\text{max}_{[\text{F16BP}]}$	0.520			
$K_{m_{[\text{F16BP}]}}$	−0.752			
μ_c	−0.826	−0.581		
a_n		−0.862		
μ_n		0.869		
Γ		0.894	0.670	
β_n		−0.893	−0.783	−0.642
ϵ	0.800	0.554		
η	−0.803	−0.585		
q	0.654			
λ		0.600		
$V\text{max}_{[\text{G3P}]}$	0.639			
$K_{m_{[\text{G3P}]}}$	−0.836			
$[\text{G6P}]_0$	0.761			
$[\text{F16BP}]_0$	0.812			
C_0	0.990			
T_0	0.796	−0.674		

Case of No RdCVF

Table 18: PRCC Data for parameters with |PRCC| > 0.5 and p-value < 0.05. **No RdCVF**. These PRCC values correspond to Figure 9 in the main text.

Parameter	PRCC 60 Min	PRCC Day 1	PRCC Day 7	PRCC Day 14
α	0.611			
$K_{m_{\text{[F16BP]}}}$		-0.627	-0.644	
μ_c	-0.844	-0.855	-0.758	-0.727
a_n		-0.868	-0.765	-0.756
μ_n		0.841		
Γ		-0.668	-0.671	
ϵ	0.840	0.816	0.692	0.706
η	-0.545	-0.830	-0.691	-0.692
q	0.553	0.728		
$V\text{max}_{\text{[G3P]}}$	0.550	0.707		
$K_{m_{\text{[G3P]}}}$	-0.793	-0.891		
$[\text{G6P}]_0$		0.644	0.780	0.733
$[\text{F16BP}]_0$	0.784	0.896		
C_0	0.815			
R_{n_0}		0.515		
T_0	0.815			
$[\text{G3P}]_0$	0.631			
